# Jiedu Sangen Decoction Reverses Epithelial-to-mesenchymal Transition and Inhibits Invasion and Metastasis of Colon Cancer via AKT/GSK-3β Signaling Pathway

**DOI:** 10.7150/jca.32873

**Published:** 2019-10-20

**Authors:** Li Yuan, Kai Zhang, Meng-Meng Zhou, Harpreet S. Wasan, Fang-Fang Tao, Qing-Ying Yan, Guan Feng, Yin-Shan Tang, Min-He Shen, Sheng-Lin Ma, Shan-Ming Ruan

**Affiliations:** 1 The First Clinical Medical College of Zhejiang Chinese Medical University, Hangzhou, 310053, Zhejiang, china; 2 Department of Medical Oncology, The First Affiliated Hospital of Zhejiang Chinese Medical University, Hangzhou, 310006, Zhejiang, China; 3 Department of traditional Chinese medicine, The First people's Hospital of Quzhou, 324000, Zhejiang, China; 4 Department of Cancer Medicine, Hammersmith Hospital, Imperial College Healthcare NHS Trust, London, W12 0HS, UK; 5 Department of Immunology and Microbiology, Basic Medical College, Zhejiang Chinese Medical University, Hangzhou, 310053, Zhejiang, China; 6 Department of Rehabilitation in Traditional Chinese Medicine, The Second Affiliated Hospital of Zhejiang University School of Medicine, Hangzhou, 310009, Zhejiang, China; 7 Department of Oncology, The Forth Affiliated Hospital of Zhejiang Chinese Medical University, Hangzhou, 310006, Zhejiang, China

**Keywords:** Colon Cancer, Jiedu Sangen Decoction (JSD), Epithelial-mesenchymal transformation (EMT), invasion, metastasis, AKT/GSK-3β signaling pathway

## Abstract

**Ethnopharmacology relevance:** Jiedu Sangen Decoction (JSD), an empirical prescription of Traditional Chinese Medicine (TCM), has been reported to inhibit invasion and metastasis of colon cancer in our previous study. The aim of this study was to investigate the mechanism of JSD-triggered inhibition of invasion and metastasis in colon cancer.

**Methods:** *In vitro*, AKT1 knockdown (si-AKT1) or overexpression (oe-AKT1) cells were successfully constructed both in SW480 and SW620 cell lines. Si-AKT1 and oe-AKT1 cells were then treated with or without JSD. Cell invasion, metastasis potential and expression of epithelial-mesenchymal transformation (EMT)-related and AKT1/GSK-3β proteins were then observed by wound healing, transwell, and western blot assays. *In vivo*, liver metastasis model mice were developed by inoculating SW480 cells. After JSD diet intervention, living fluorescence imaging and weight measurements were carried out to investigate JSD induced inhibition effects on liver metastasis of colon cancer. Immunohistochemistry and western blot assays were performed to observe tissue features and detect protein expression.

**Results:** Invasion and metastasis potential, as well as EMT of colon cancer, can be markedly inhibited by JSD treatment or AKT1 knockdown, while enhanced by AKT1 overexpression. JSD-induced inhibition effects were significantly weakened when AKT1 was knocked down, while clearly enhanced when AKT1 was overexpressed. Additionally, JSD could lead to an increase in expression of E-cadherin, and a decrease in expression of N-cadherin, Vimentin, p-AKT1, AKT1, p- GSK-3β, Snail, Slug, and Twist in colon cancer cells.

**Conclusion:** JSD reverses EMT and inhibits invasion and metastasis of colon cancer through the AKT/GSK-3β signaling pathway.

## 1. Introduction

Colorectal cancer (CRC) is one of the most common malignant tumors of the digestive tract 1. The 5-year survival rate for stage I CRC patients can reach as high as 90%, while stage III and IV CRC patients with accompanying distant metastasis have a 5-year survival rate of just over 10%^2^. Therefore, discovering safe and effective drugs aiming at inhibiting CRC invasion and metastasis is of particular importance. Epithelial-mesenchymal transformation (EMT) refers to the biological process by which epithelial cells lose their polarity and become interstitial cells^3^. Accumulated evidence has demonstrated that EMT is closely associated with the progression of colon cancer. For example, a previous study has found that the expression of EMT-related proteins Snail and Vimentin are upregulated, while E-cadherin (E-cad) and claudin expression are significantly downregulated in intestinal mucosa tissue in patients with colon cancer^4^. Furthermore, the upregulation of E-cad and the downregulation of zinc-finger E-box binding homeobox 1 (ZEB1) and N-cadherin (N-cad) can significantly inhibit metastasis and invasion of colon cancer cells^5^. The above discoveries suggest that EMT may be a crucial process allowing for colon cancer cells to become invasive and metastasize to distant regions of the body.

AKT is a serine/threonine kinase and a central node in downstream cell signaling, controlling multiple biological processes such as cell apoptosis, proliferation, invasion, metastasis, and angiogenesis^6^. Abnormal activation of AKT commonly leads to the development of tumors^7^, while the targeted inhibition of AKT can suppress the proliferation, invasion and metastasis of tumor cells^8^. GSK-3β, the downstream molecule of AKT, participates in cell differentiation, proliferation, survival and apoptosis, and cooperates with AKT to regulate the development of EMT^9^. In esophageal cancer cells, activation of the AKT/GSK-3β pathway has been shown to enhance Snail expression and promote the occurrence of EMT^10^. A similar result is observed in lung cancer cells, where inhibition of the AKT/GSK-3β pathway can induce the downregulation of Slug, N-cad, and Vimentin, thus reversing EMT^11^. However, whether the AKT/GSK-3 β signaling pathway is a key mechanism involved in the process of EMT in CRC has not yet been documented.

Traditional Chinese Medicine (TCM) is systematically developed through thousands of years of continuous refinement and practice. Based on the unique advantage that TCM has to reduce the toxic side effects of tumor treatment, improve living quality and extend life-span, etc., TCM has currently been an important part of anti-tumor treatment. Jiedu Sangen Decoction (JSD) is an empirical prescription that has been used for colon cancer treatment since the 1970s by Traditional Chinese Medical Hospital of Zhejiang Province, which is made from *Actinidia argute Siebold & Zucc.*, *Adina fauriei H.Lév.*, and *Polygonum cuspidatum Siebold & Zucc*.^12^, ^13^. Our previous studies have revealed that JSD can inhibit the invasion and metastasis of colon cancer cells^12^, ^13^. Therefore, it is meaningful to investigate the mechanism of JSD-triggered inhibition effects on colon cancer invasion and metastasis. The present study may provide a novel perspective for TCM treatment in malignant tumors.

## 2. Materials and methods

### 2.1 Cell culture

SW480, SW620 and HCT-8 cells were purchased from a typical culture preservation commission cell bank, Chinese Academy of Sciences (Shanghai, China). SW480 and HCT-8 cells were cultured in RPMI 1640 medium (Kino biological and Pharmaceutical Technology Co., Ltd, Hangzhou, China) containing 10% fetal bovine serum (FBS; Gibco, Grand Island, USA) and 1% penicillin/streptomycin (Kino Co., Ltd, Hangzhou, China) at 37°C under 5% CO_2_ in a cell culture incubator. SW620 cells were cultured in L-15 medium (Kino Co., Ltd, Hangzhou, China) containing 10% FBS and 1% penicillin/streptomycin in a sealant culture bottle at 37°C under 5% CO_2_ conditions.

### 2.2 Preparation of JSD

The medicinal materials of JSD including *Actinidia argute Siebold & Zucc.* (Cat No. 20160721), *Adina fauriei H.Lév.* (Cat No. 20160810) and *Polygonum cuspidatum Siebold & Zucc.* (Cat No. 20160702) were purchased from the pharmacy of Zhejiang Provincial Hospital of TCM (Zhejiang, China) (Figure S1A). The place of origin of all three materials is Zhejiang. *Actinidia argute* Siebold & Zucc. (100 g), Adina fauriei H.Lév. (100 g), and *Polygonum cuspidatum* Siebold & Zucc. (100 g) were mixed and immersed in 1000 mL of distilled water for 30 min. The filtrates were concentrated to 150 mL to obtain JSD such that its crude drug content was 2 g/mL of mother liquor. The JSD was prepared at the China Pharmaceutical University (Jiangsu, China). Through the information analysis of HPLC-UV and HPLC-MS for JSD (Figure S1 B-C), the quality control indicator is confirmed as di-(2-ethylthexyl) phthalate for *Actinidia argute Siebold & Zucc.*, 5-hydroxyl-2-methyl chromocone-7-O-beta-D-celery sugar (1 to 6)-beta-D-glucoside for *Adina fauriei H.Lév.*, and Resveratrol-3-O-glucoside for *Polygonum cuspidatum Siebold & Zucc.* (Table S1). After comparative analysis of the HPLC-UV from 3 batches of JSD, we confirmed that the composition of JSD is stable and can be used for our experiments (Figure S1D).

### 2.3 EGF induced EMT model

For EGF treatment^14^-^16^, SW480, SW620 and HCT-8 cells in logarithmic phase were harvested and seeded into culture bottles at a density of 1×10^6^ per bottle. After cell attachment, epidermal growth factor (EGF; R&D Systems, Minneapolis, MN, USA) at a final concentration of 50 ng/ml was added to each bottle for 48 h. To verify success of EGF-induced EMT, the expression of E-cad, N-cad and Vimentin were detected by WB (Figure S1E-H).

### 2.4 Cell viability assay

Cell proliferation was evaluated by the Cell Counting Kit-8 (CCK-8) assay (GEN biotech, Jiangsu, China). Cells were seeded in 96-well plates (1×10^4^ cells/well.). When the cells grew to a confluence of 60%, the culture medium was replaced with JSD at different concentrations. After 48 h of incubation, CCK-8 reagent was added to culture medium and incubated with the cells for an additional 2 h. Absorbance was measured by a microplate reader (Biorad, USA) at 450 nm. The proliferation inhibition rates (%) = (the average OD value in all duplicates in control group - the average OD value in medicine groups) / the average OD value in blank control group*100%.

### 2.5 Wound Healing Assay

For wound-healing assays, approximately 2×10^5^ SW480, SW620 or HCT-8 cells were seeded onto 6-well plates. After cell attachment, EGF at a final concentration of 50 ng/ml was added to cells in each well for 48 h to induce EMT. Cells then received 6 mg/ml JSD intervention for 48 h. Finally, three fields of vision were randomly selected for each group and photos were taken at 100× magnification under the optical microscope (ix71, Olympus, Japan) at 0 h and 24 h after wound induction.

### 2.6 Transwell migration and invasion (Matrigel) experiments

In transwell migration experiments, SW480, SW620 and HCT-8 cells, with and without JSD treatment, were digested and collected. Cell density was adjusted to 2×10^6^/ml. For each group, 200 μl cell suspension in RPMI 1640 medium (serum-free) was added to the upper chamber (24-well, Corning, New York, NY, USA), and 600 μl serum-containing RPMI 1640 medium was added to the lower chamber. After incubation for 48 h, invaded cells were fixed with 4% paraformaldehyde (Boster Company, Wuhan, China) for 20 min and then stained with 0.1% crystal violet (Shanghai Sangon, China) for 30 min. Finally, photos were taken and the number of invaded cells were counted by using a light microscope (400×) (ix71, Olympus, Japan). In transwell invasion experiments, 40 μl diluted Matrigel glue (Corning, New York, NY, USA) was evenly spread on the upper chamber (24-well) and allowed to solidify. The remaining steps were the same as the transwell migration experiments.

### 2.7 Real-time Quantitative PCR (RT-qPCR)

Total RNA was extracted by TRIzol reagent (Shanghai Pufei Biotech Co., Ltd, Shanghai, China) from cells of each group. cDNA was synthesized by using M-MLV Reverse Transcriptase kit (Promega, Madison, WI, USA). For two-step RT-qPCR, each reaction was run in 12 μl reaction mixture containing 0.6 μl of template cDNA, 0.3 μl of primer mix (5 μM), 6 μl SYBR premix ex taq (Takara Bio, Shiga, Japan) and 5.1 μl RNase-Free H_2_O. Primers synthesized by Shanghai Sangon Biological Engineering and Technology Service (Shanghai, China) are listed as follows: AKT1 (287bp): 5'-GTG CTG GAG GAC AAT GAC TAC-3' (Forward), 5'-TGC TGC CAC ACG ATA CCG-3' (Reverse); GAPDH (121bp): 5'-TGA CTT CAA CAG CGA CAC CCA-3' (Forward), 5'-CAC CCT GTT GCT GTA GCC AAA-3' (Reverse). The relative level of each gene was calculated according to the following formula: 2^-ΔΔCt^=2-^[ΔCt(objective gene)-ΔCt(GAPDH)]^).

### 2.8 Western Blot

Cell or tissue protein lysates were separated in 10% SDS-polyacrylamide gels and then transferred to a PVDF membrane (Millipore, MA, USA). Target proteins were probed with primary antibodies at 4 °C overnight. Membranes were then incubated with a corresponding horseradish peroxidase (HRP)-conjugated secondary antibody at room temperature for 1 h. Finally, the bands were visualized by enhanced chemiluminescence (ECL; Thermo-Pierce, Rockford, IL, USA). Image-Pro Plus Version 6 software was used to analyze the integral optical density (IOD) value. Data were calculated and normalized to β-actin. Antibodies are listed as follows: N-Cad (Cat#76011, 1:500), E-Cad (Cat#76319, 1:1000), Vimentin (Cat#5741, 1:1000), AKT1 (Cat#4691, 1:1000), p-AKT (Ser473) (Cat#4060, 1:1000), p-GSK-3β (Ser9) (Cat#5558, 1:1000), GSK-3β (Cat#12456, 1:1000), Snai1 (Cat#3879S, 1:500), SNAI2 (Slug) (Cat#27568, 1:500), Twist1 (Twist) (Cat#175430, 1:1000), β-actin (Cat#20536-1-AP, 1:1000), Peroxidase Conjugated Goat anti-Mouse IgG (H+L) (Cat#DW0990, 1:1000), Peroxidase Conjugated Goat anti-Rabbit IgG (H+L) (Cat#DW-GAR007, 1:1000).

### 2.9 Establishment of stable cell lines of AKT1 knockdown (si-AKT1) and AKT1 overexpression (oe-AKT1)

Human AKT1-shRNA and AKT1-overexpression lentiviral vectors were constructed, identified and supplied by Shanghai Genechem Chemical Technology Co., Ltd (Genechem, Shanghai, China). For *AKT1* silencing, among three designed AKT1 siRNA target sequences tested, the sequence with the best silencing efficiency was: 5'-GAT CCT CAA GAA GGA AGT CAT-3'. After annealing, oligonucleotides were cloned into the AgeI/EcoRI sites of GFP-tagged GV248 lentivirus vectors (Genechem, Shanghai, China). After identification of the correct sequence and lentivirus packaging, SW480 and SW620 cells were infected at MOI=10 for 16 h. For AKT1 overexpression, the cDNA of AKT1 was sub-cloned using Taq DNA polymerase (SinoBio Biltech Co. Ltd, Shanghai, China) and inserted into the BamHI/AgeI sites of GFP-tagged GV260 lentivirus vectors (Genechem, Shanghai, China). After identification of the correct sequence and lentivirus packaging, SW480 and SW620 cells were infected at MOI=10 for 12 h. AKT1 knockdown (si-AKT1) or overexpression (oe-AKT1) cells were successfully constructed both in SW480 and SW620 lines. In the HCT-8 cell line, we did not succeed in establishing AKT1 knockdown (si-AKT1) or overexpression (oe-AKT1) cells. Therefore, only SW480 and SW620 cell lines were selected for subsequent experiments.

### 2.10 Liver metastasis of colon cancer in nude mice

All animal experiments were approved by the Institutional Animal Care and Use Committee of Zhejiang Chinese Medical University. Mice were fed in the Specific Pathogen Free (SPF) barrier center at the animal experimental center of Zhejiang Chinese Medical University, under standard conditions of temperature (25 ± 2 °C) and humility (50 ± 5%) in a 12 h light/12 h dark cycle with normal drink and food.

SW480 cells from si-AKT1, oe-AKT1 and corresponding NC groups were collected. Cell concentration was adjusted to 3×10^7^/ml. To establish a liver metastasis model of colon cancer, each nude mouse was inoculated with 0.1 ml of the above cell suspension in their spleen membrane^17^-^19^.The conditions of the animal model were evaluated by living fluorescence imaging technique after two weeks. According to total flux *in vivo* imaging, the eligible mice were divided into blank control (NC-control, si-AKT1-control, oe-AKT1-control) and JSD (NC-JSD, si-AKT1-JSD, oe-AKT1-control) groups. Mice in the JSD groups were fed with JSD (1.2 g/ml, 0.4 ml/20g) one time per day lasting for 2 weeks. Mice in blank control groups were given normal saline one time per day for 2 weeks. Then, liver metastasis was observed by living fluorescence imaging technique. On day 28, mouse weight was recorded and the liver, spleen and tumor foci were collected.

### 2.11 Bioluminescence imaging

*In vivo* bioluminescence imaging was carried out by using a cooled CCD camera system (IVIS Imaging System, PerkinElmer, CA, USA). Briefly, normal saline containing 15 mg/mL D-luciferin (Art.No.40901ES03, Yeasen Corp., Shanghai, China) was intraperitoneally injected into mice per 150 mg/kg body weight. These mice were placed in the light-tight chamber of the CCD camera system accompanying 2% isoflurane anesthesia. For luminescent image acquisition, an integration time of 1 to 60 sand binning factors of 4 was used. Signal intensity was measured according to flux of all detected photon counts from the region of interest prescribed over tumor area using the Living Image software package (Xenogen Corp., Alameda, CA, USA).

### 2.12 Immunohistochemistry

For immunohistochemistry assays, after treating with 3% H_2_O_2_/methyl alcohol solution for 10 min at room temperature, 5% normal goat serum buffer was used to block the tissue at 37 ℃ for 30 min. Slides were then incubated with primary antibodies at 4 ℃ overnight. After washing, the slides were incubated with biotin labeled goat anti-rabbit IgG and HRP-conjugated streptavidin at 37℃ for 1 h. Immunoreaction was visualized by diaminobenzidine (DAB) (Cat#ZLI-9065, ZSGB-BIO Corp., Shanghai, China). After DAB staining, all tissues were counterstained with hematoxylin (Cat#ZLI-9609 ZSGB-BIO Corp., Shanghai, China) dehydrated and then blocked. The results were analyzed and photographed with an inverted microscope at 200× magnification. The total integral optical density (IOD) of positive area was analyzed by Image-Pro Plus Version 6 software. The concentration of antibodies used were as follows: N-Cad (1:300), E-Cad (1:300), Vimentin (1:300), Hypersensitive enzyme labeled Goat anti mouse/rabbit IgG polymer (Cat#ZB-2305, ZSGB-BIO Corp., Shanghai, China)(1:1).

### 2.13 Statistical analysis

All measurements are represented as Mean ± standard deviation (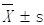) and analyzed with SPSS statistics version 22.0 (IBM, New York, NY, USA). All experiments were repeated in triplicate. One-way ANOVA was performed when data followed a normal distribution. Nonparametric test was performed when data did not follow a normal distribution. A probability value of *P*<0.05 was considered statistically significant.

## 3. Result

### 3.1 JSD-induced inhibition effects on proliferation of colon cancer cells *in vitro*

**Figure 1:**
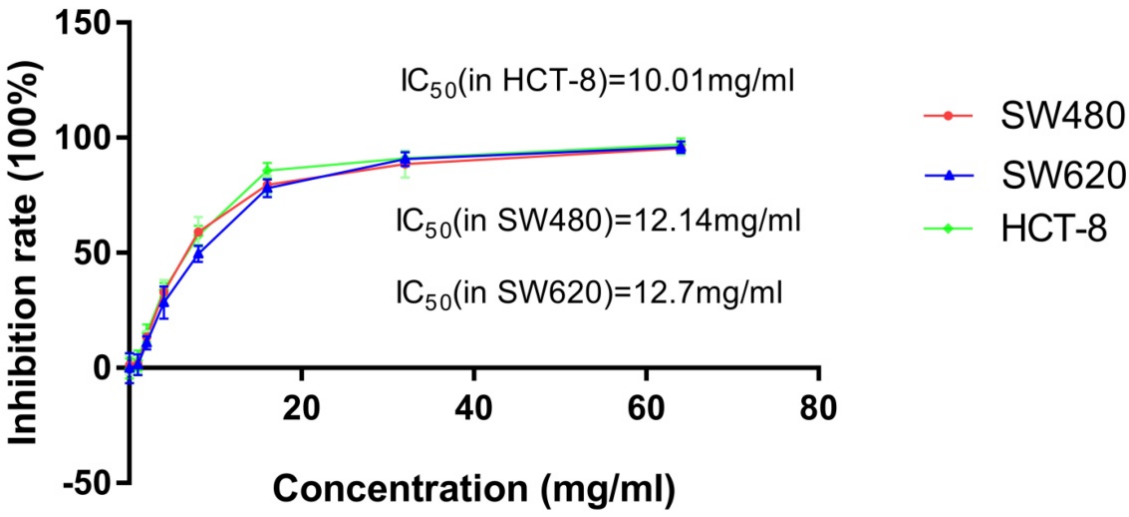
CCK-8 assay showed the cytotoxic effect of JSD on inhibition rate of cell proliferation in SW480, SW620 and HCT-8 cells. Cells were treated with JSD at the concentrations of 0, 1mg/ml, 2mg/ml, 4mg/ml, 8mg/ml, 16mg/ml, 32mg/ml, or 64mg/ml. Inhibitory effects were increased with the increase of JSD concentration. The half inhibition concentration (IC50) of JSD was 12.14mg/ml in SW480 cells, 12.7mg/ml in SW620 cells and 10.01mg/ml in SW620 cells; n=9.

To observe the inhibitory effect of JSD on the proliferation of colon cancer cells, SW480, SW620 and HCT-8 cells were treated with increasing concentrations (0, 1mg/ml, 2mg/ml, 4mg/ml, 8mg/ml, 16mg/ml, 32mg/ml, 64mg/ml) of JSD for 48 h. As shown in Figure 1 and Table 1, inhibition of SW480, SW620 and HCT-8 cell proliferation positively correlated with JSD concentration. The IC_50_ of JSD was 12.14 mg/ml in SW480 cells, 12.7 mg/ml in SW620 cells and 10.01 mg/ml in HCT-8 cells. Therefore, 6 mg/ml was used for our following experiments to observe the effect of JSD on invasion and metastasis of colon cancer cells.

### 3.2 JSD-induced inhibitory effects on the migration and invasion potential of colon cancer cells *in vitro*

**Figure 2:**
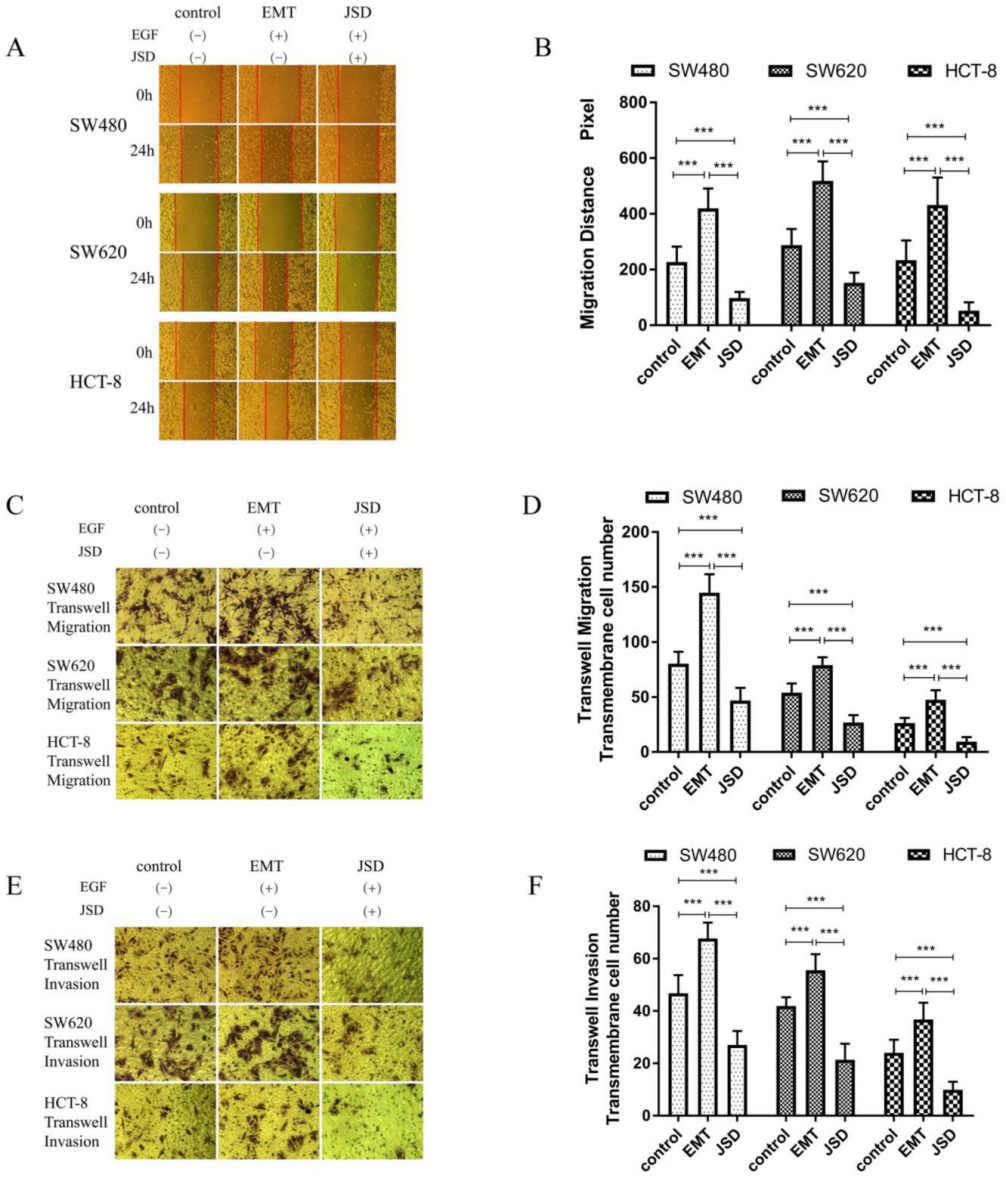
The JSD-induced inhibitory effects on the migration and invasion potential of colon cancer cells *in vitro*. The experimental group consisted of control, EMT, and JSD treated cells. The EMT and JSD groups were treated with 50 ng/mL EGF for 48 h. Next, the JSD group was treated with 6 mg/mL JSD for 48 h. (A-B): Compared with the control group, the migration distance of the EMT group was significantly increased (both, P<0.001), while the migration distance of the JSD group was decreased (both, P<0.001). In addition, the migration distance of the JSD group was significantly shorter than that in the EMT group (both, P<0.001). (C-F): the number of transmembrane cells in the EMT group was enhanced in comparison with that in the control group (all, *P<*0.001). Conversely, the number of transmembrane cells in the JSD group was significantly less in comparison with the control group (both, *P<*0.001). Compared with the EMT group, the number of transmembrane cells in the JSD group was also significantly reduced (all, *P<*0.001) compared with the respective control, ***P<0.001; n=9.

Wound healing assays were performed with SW480, SW620 and HCT-8 cells in three groups (control, EMT and JSD). The EMT and JSD treated groups received EGF at a final concentration of 50 ng/ml for 48 h to induce EMT. The JSD group received 6 mg/ml JSD intervention for an additional 48 h. As shown in Figure 2A and 2B, the migration distance of the EMT group was significantly increased when compared with the control group (all, P<0.001), and the migration distance of the JSD group was decreased (all, P<0.001). In addition, the migration distance of the JSD group was significantly shorter than that of the EMT group (all, P<0.001).

Similar to the results seen in the wound-healing assays, transwell migration and invasion assays revealed that the number of transmembrane cells in the EMT group was increased in comparison with that in the control group (all, P<0.001; Figure 2C-2F) and the number of transmembrane cells in the JSD group was significantly decreased in comparison with the control group (all, P<0.001). Furthermore, compared with the EMT group, the number of transmembrane cells in the JSD group was significantly reduced (all, P<0.001). Taken together, these results suggest that JSD can inhibit migration and invasion as well as reverse the EMT status of colon cancer cells.

### 3.3 JSD-induced reversion of EMT depends on activation of AKT/GSK-3β signaling *in vitro*

**Figure 3:**
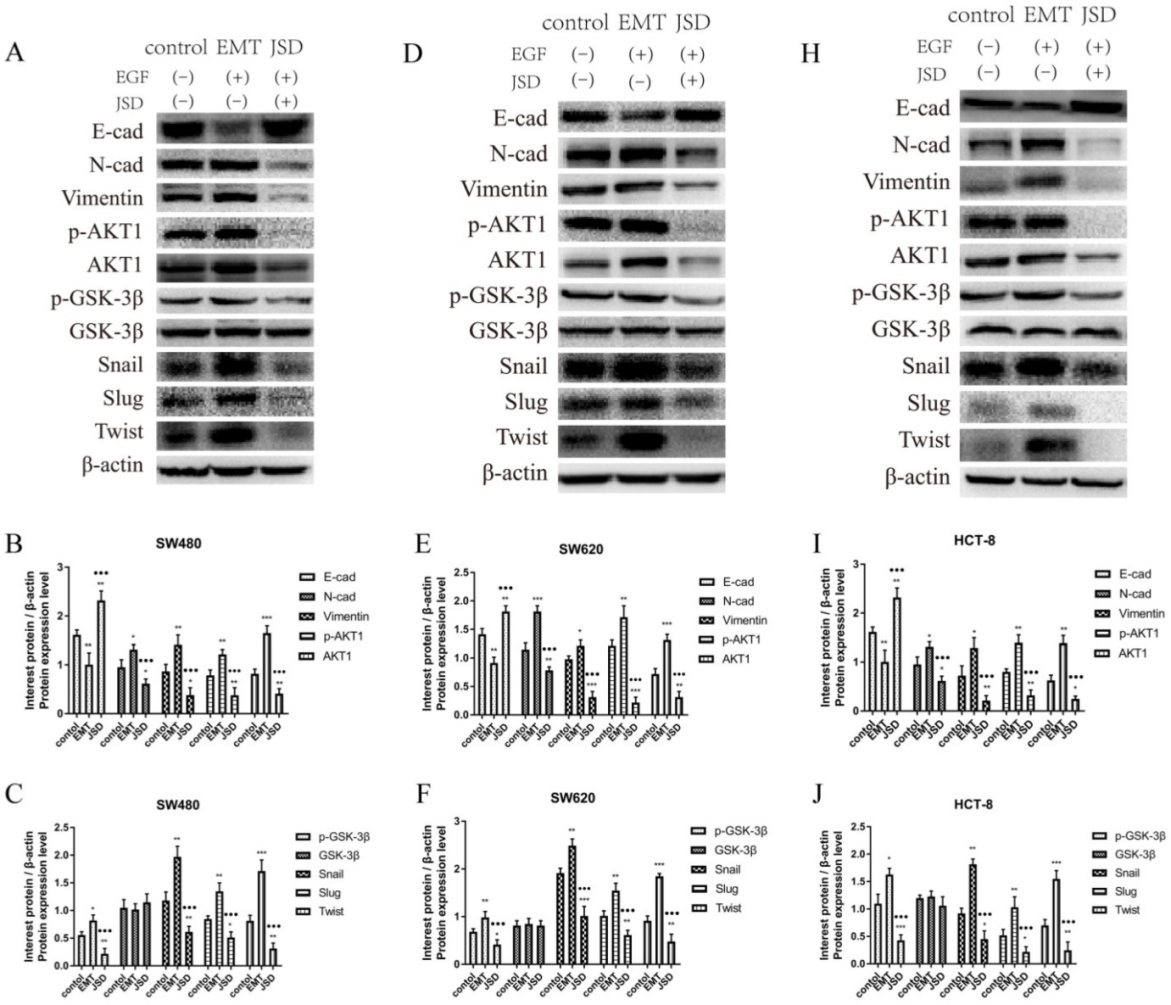
JSD-induced reversion on EMT progression of colon cancer cells *in vitro.* The experimental groups consisted of control, EMT, and JSD treated. The EMT and JSD groups were treated with 50 ng/mL EGF for 48 h. Next, the JSD group was treated with 6 mg/mL JSD for 48 h. (A) Western blot assays in SW480 cells. (B) and (C) Relative expression of indicated proteins in SW480 cells. (D) Western blot assays in SW620 cells. (E) and (F) Relative expression of indicated proteins in SW620 cells. (H) Western blot assays in HCT-8 cells. (I) and (J) Relative expression of indicated proteins in HCT-8 cells. Compared to the control group, the expression of E-cad was decreased, while N-cad, Vimentin and EMT related transcription factors Snail, Slug, Twist were increased in the EMT group (all, P<0.05). Additionally, the p-AKT1, AKT1 and p-GSK-3β levels in the EMT group were higher than those in the control group (all, P<0.05). On the contrary, E-cad was significantly upregulated in the JSD group in comparison with the control and EMT groups (all, P<0.05), while N-cad, Vimentin, p-AKT1, AKT1, p-GSK-3β, Snail, Slug and Twist were downregulated (all, P<0.05). Compared with NC-control group, *P<0.05, **P<0.01, ***P<0.001; compared with EMT group, ●●●*P*<0.001. n=3.

To explore the potential mechanism of JSD treatment on the progression of EMT, EMT related proteins and AKT/GSK-3β signaling pathway proteins were analyzed by western blot. The results revealed that compared to the control group, the expression of E-cad was decreased, while N-cad, Vimentin and EMT related transcription factors Snail, Slug, and Twist were increased in the EMT group (all, P<0.05; Figure 3). Additionally, the p-AKT1, AKT1 and p-GSK-3β levels in the EMT group were higher than those in the control group (all, P<0.05). On the contrary, E-cad was significantly upregulated in the JSD group in comparison with that of the control and EMT groups (all, P<0.05), and N-cad, Vimentin, p-AKT1, AKT1, p-GSK-3β, Snail, Slug and Twist were all downregulated (all, P<0.05). The results suggest that JSD might downregulate the EMT transcription factors Snail, Slug and Twist, leading to the upregulation of E-cad and downregulation of N-cad and Vimentin. This change in protein expression leads to a reverse in the EMT status of colon cancer cells, presumably through the AKT/GSK-3β signaling pathway.

### 3.4 Knockdown of AKT1 weakened JSD-induced inhibition on the migration and invasion potential of colon cancer cells *in vitro*

**Figure 4:**
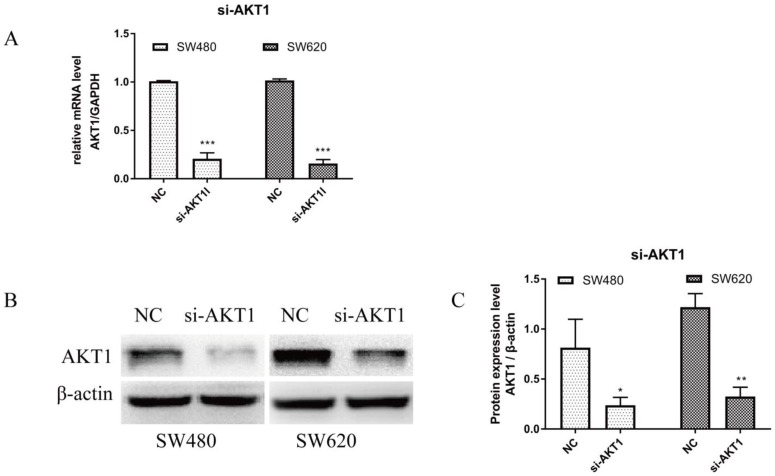
AKT1 was successfully knocked down in SW480 and SW620 cells. (A): Real-time quantitative PCR (qPCR) showed that the mRNA expression of AKT1 in SW480 and SW620 cells transfected with si-AKT1 was significantly decreased compared with that in the two cell lines transfected with NC. (B-C): Western blot analysis showed that the protein expression of AKT1 in SW480 and SW620 cells transfected with si-AKT1 was significantly decreased compared with that in the two cell lines transfected with NC. Compared with NC group, *P<0.05, **P<0.01, ***P<0.001; n=3.

RT-qPCR and WB were performed on cells transfected with si-AKT1 to confirm successful silencing. As shown in Figure 4A, AKT1 mRNA levels were significantly lower in si-AKT1 transfected cells than that of the NC group (both, P<0.001). Furthermore, the protein expression of AKT1 was significantly decreased in si-AKT1 cells when compared to the NC group, indicating successful gene silencing (Figure 4B and 4C; SW480, P<0.05; SW620, P<0.01).

**Figure 5:**
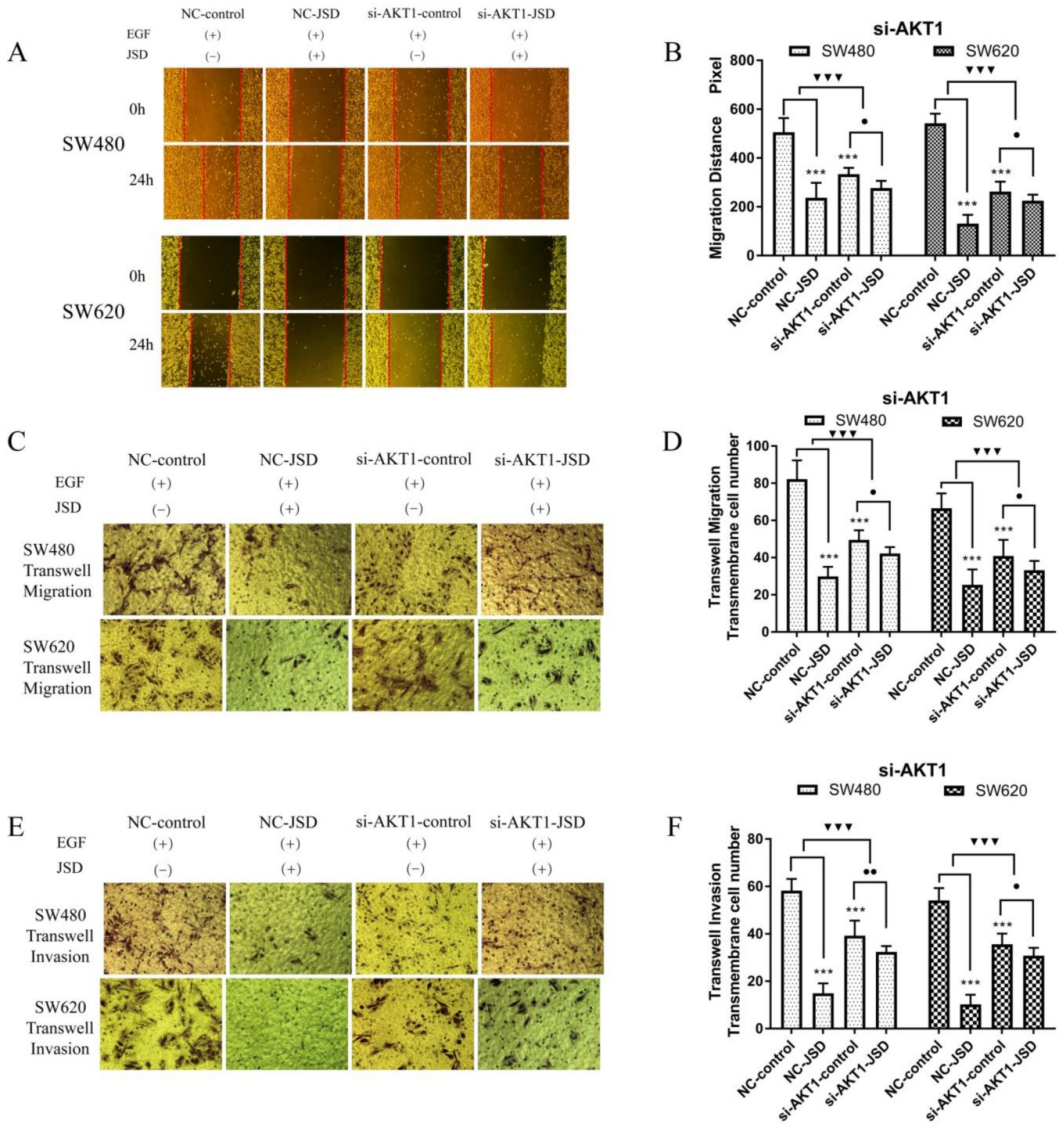
Knockdown of AKT1 weakened the effects of JSD-induced inhibition on the migration and invasion potential of colon cancer cells *in vitro.* The experiment consisted of four groups (NC-control, NC-JSD, si-AKT1-control and si-AKT1-JSD group). All groups received EGF at a final concentration of 50 ng/ml for 48 h to induce EMT. NC-JSD and si-AKT1-JSD groups to receive 6 mg/ml JSD intervention for an additional 48 h. (A-B): The 24 h migration distance of both NC-JSD groups was significantly shorter than that of the NC-control groups. The 24 h migration distance of si-AKT1-control cells was also significantly shorter than that of cells in the NC-control group. 24 h migration distance of cells in the si-AKT1-JSD group was also shorter than that of cells in the si-AKT1-control group, but the difference between the si-AKT1-control and si-AKT1-JSD groups was smaller than that seen in the NC-control and NC-JSD groups. (C-F): The number of migrated and invaded cells in the NC-JSD group was significantly lower than that in the NC-control group. In addition, the number of migrated and invaded cells in the si-AKT1-control group was significantly lower than that in the NC-control group. The number of migrated and invaded cells in the si-AKT1-JSD group was also lower than that in cells of the si-AKT1-control group, however, the difference between the si-AKT1-control and si-AKT1-JSD groups was smaller than that between the NC-control and NC-JSD groups. Compared with NC-control group, ***P<0.001; compared with si-AKT1-control group, ●*P*<0.05, ●●*P*<0.01; compared between NC-groups and si-AKT1-groups, ▼▼▼*P*<0.001; n=9.

Wound healing assays were performed in SW480 and SW620 cells for four cell groups (NC-control, NC-JSD, si-AKT1-control and si-AKT1-JSD). All groups received EGF at a final concentration of 50 ng/ml for 48 h to induce EMT. NC-JSD and si-AKT1-JSD groups received 6 mg/ml JSD treatment for an additional 48 h. As shown in Figure 5A-5B, we observed a decrease in 24 h migration distance in both NC-JSD cell lines when compared to NC-control groups (both, P < 0.001), which indicated that JSD inhibited the healing ability of colon cancer cells. The 24 h migration distance of cells in the si-AKT1-control group was also significantly shorter than that in cells of the NC-control group (both, P < 0.001), indicating that knockdown of AKT1 was also sufficient to inhibit the healing ability of colon cancer cells. Additionally, the 24 h migration distance of si-AKT1-JSD cells was shorter than that of cells in the si-AKT1-control group (both, P < 0.05). However, the difference between the migration distances of the two si-AKT1 groups was smaller than that between the NC-control and NC-JSD groups (both, P < 0.001), suggesting that the ability of JSD to inhibit the healing ability of colon cancer cells was weakened after AKT1 knockdown.

Transwell assays were performed to assess the migration and invasion potential of both cell lines, with drug treatment the same as that done in the wound healing assays (Figure 5C-5F). NC-JSD cells had a significantly reduced migration and invasion potential compared with NC-control cells (all, P < 0.001), indicating that JSD dramatically inhibited the migration and invasion of colon cancer cells. The number of migrated and invaded cells in the si-AKT1-control group was also significantly lower than that in the NC-control group (all, P < 0.001), indicating that AKT1 knockdown also reduced the migration and invasion potential of colon cancer cells. Furthermore, the number of migrated and invaded cells in si-AKT1-JSD cells was lower than that of si-AKT1-control cells (all, P < 0.05). However, the difference between the si-AKT1-control and si-AKT1-JSD groups was smaller than that seen in the NC-control and NC-JSD groups (all, P < 0.001), suggesting that the ability of JSD to inhibit migration and invasion of colon cancer cells was mitigated by AKT1 knockdown.

### 3.5 Knockdown of AKT1 weakened the effects of JSD-induced reversion of EMT *in vitro*

**Figure 6:**
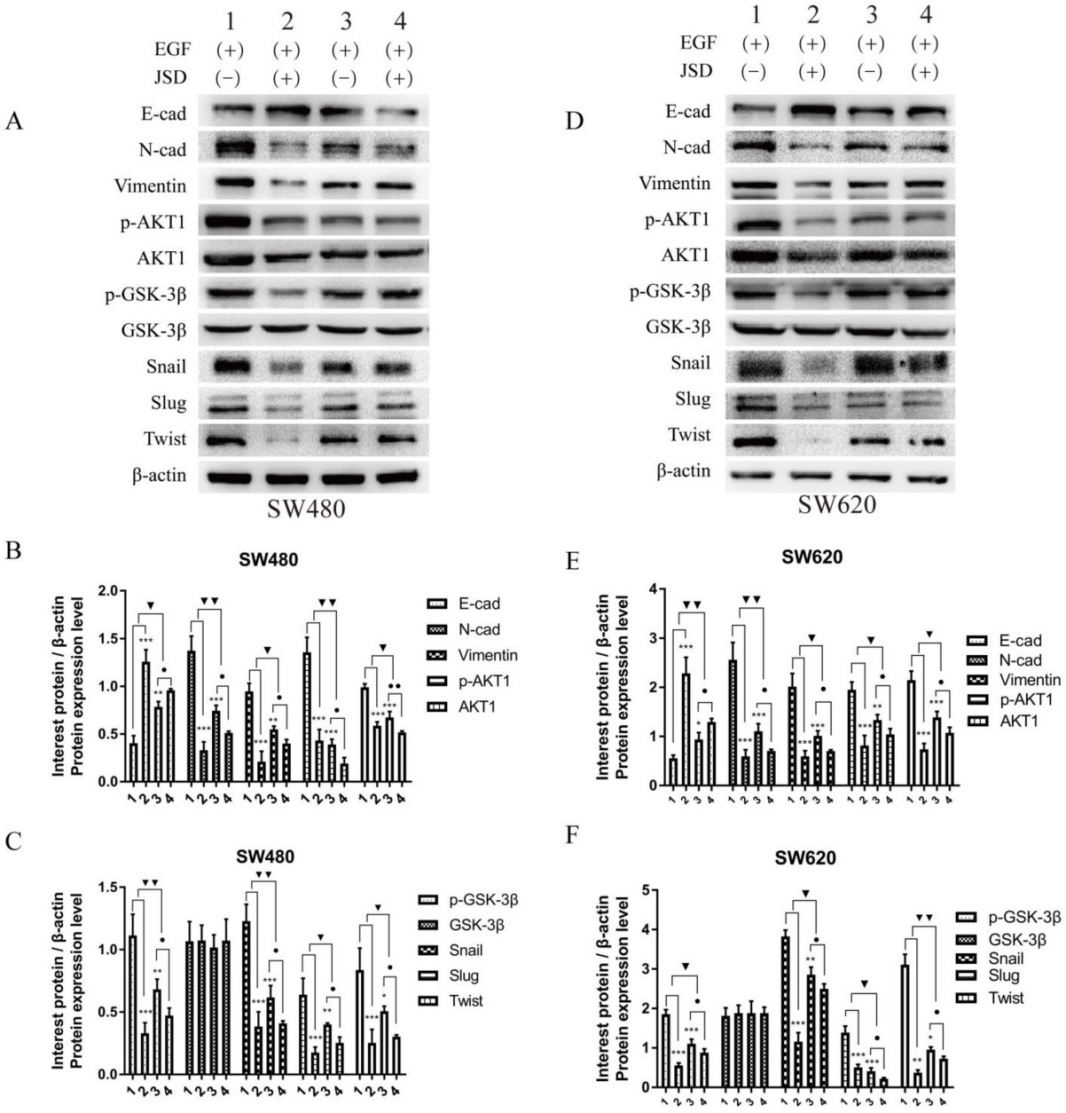
Knockdown of AKT1 weakened the effects of JSD-induced reversion of EMT *in vitro.* JSD drug treatment for Western Blot assays was similar to that done in wound healing assays. (1=NC-control, 2= NC-JSD, 3=si-AKT1-control, 4=si-AKT1-JSD group). (A-C) The expression of indicated proteins in SW480 cells. (D-F) The expression of indicated proteins in SW620 cells. The expression of E-cadherin in both cell lines was markedly upregulated in the NC-JSD and si-AKT1-control groups compared to that in the NC-control groups, whereas N-cadherin, vimentin, p-AKT1, AKT1, p-GSK-3β, GSK-3β, Snail, Slug and Twist expression was downregulated. The expression of E-cadherin in both cell lines was also upregulated in the si-AKT1-JSD groups compared to that in the si-AKT1-control groups, whereas N-cadherin, vimentin, p-AKT1, AKT1, p-GSK-3β, GSK-3β, Snail, Slug and Twist expression was markedly downregulated. However, the difference between in the si-AKT1-control and si-AKT1-JSD groups was less than that between the NC-control and NC-JSD groups. Compared with NC-control group, *P<0.05, **P<0.01, ***P<0.001; compared with si-AKT1-control group, ●*P*<0.05; compared between NC-groups and si-AKT1-groups, ▼*P*<0.05, ▼▼*P*<0.01; n=3.

Western blot assays were done to detect the expression of EMT markers and AKT/GSK-3β/Snail signaling pathway related proteins in SW480 and SW620 cell lines. JSD drug treatment for Western blot assays was similar to that done in wound healing assays. As shown in***Figure 6***, the protein expression of E-cadherin in both cell lines was markedly upregulated in NC-JSD and si-AKT1-control cells compared to NC-control cells, whereas N-cadherin, vimentin, p-AKT1, AKT1, p-GSK-3β, GSK-3β, Snail, Slug and Twist expression was markedly downregulated (all, *P* < 0.05), indicating reversal of EMT status after JSD treatment or knockdown of AKT1. The protein expression of E-cadherin in both cell lines was also upregulated in the si-AKT1-JSD cells compared to si-AKT1-control cells, whereas N-cadherin, vimentin, p-AKT1, AKT1, p-GSK-3β, GSK-3β, Snail, Slug and Twist expression was markedly downregulated (all, *P* < 0.05). However, the difference between the si-AKT1-control and si-AKT1-JSD groups was less than that between the NC-control and NC-JSD groups (all, *P* < 0.05), suggesting that the ability of JSD to reverse EMT in colon cancer cells was mitigated by AKT1 knockdown.

### 3.6 Overexpression of AKT1 enhanced the effects of JSD-induced inhibition on the migration and invasion potential of colon cancer cells *in vitro*

**Figure 7:**
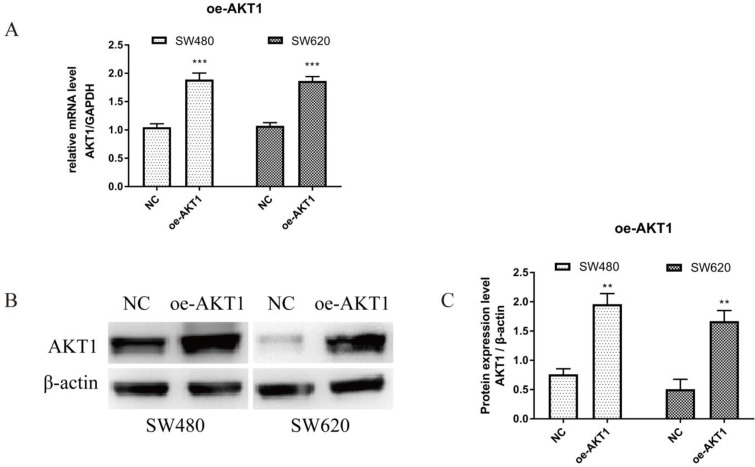
AKT1 was successfully overexpressed in SW480 and SW620 cells. (A): Real-time quantitative PCR (qPCR) showed that mRNA expression of AKT1 in SW480 and SW620 cells transfected with oe-AKT1 was significantly increased compared with that of the two cell lines transfected with NC. (B-C): Western blotting showed that the protein expression of AKT1 in SW480 and SW620 cells transfected with oe-AKT1 was significantly increased compared with that of the two cell lines transfected with NC. Compared with NC group, **P<0.01, ***P<0.001; n=3.

To verify successful transfection of AKT1, RT-qPCR and WB were performed. As shown in Figure 7A, AKT1 mRNA levels were significantly higher in SW480 and SW620 cells transfected with oe-AKT1 than that in NC cells (P<0.001). AKT1 protein expression was also significantly increased in oe-AKT1 cells when compared to NC cells (Figure 7B and 7C; SW480 and SW620, P<0.01). Taken together, these data indicate successful transfection and expression of AKT1 in both cell lines.

**Figure 8:**
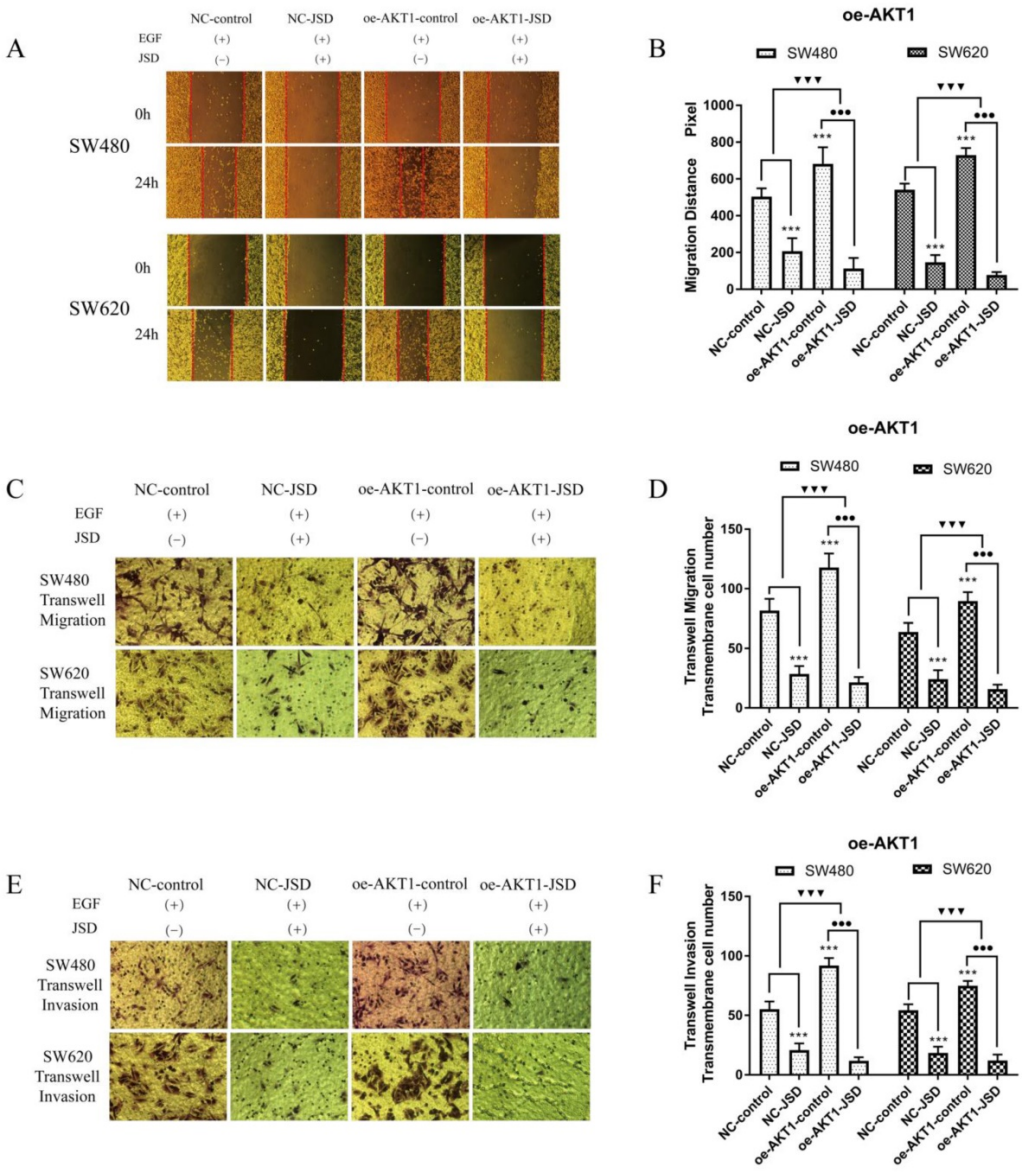
Overexpression of AKT1 enhanced the effects of JSD-induced inhibition on migration and invasion potential of colon cancer cells *in vitro.* The experiment consisted of four groups (NC-control, NC-JSD, oe-AKT1-control and oe-AKT1-JSD). (A-B): The 24 h migration distance of both cell lines in the NC-JSD group was significantly shorter than that in NC-control groups. The 24 h migration distance of cells in the oe-AKT1-control group was significantly longer than that in cells of the NC-control group. The 24 h migration distance of cells in the oe-AKT1-JSD group was also significantly shorter than that in cells of the oe-AKT1-control group. Moreover, the difference between the oe-AKT1-control and oe-AKT1-JSD groups was larger than that between the NC-control and NC-JSD groups. (C-F): The number of migrated and invaded cells in the NC-JSD group was significantly less than that in the NC-control group. In addition, the number of migrated and invaded cells in the oe-AKT1-control group was significantly more than that in the NC-control group. The number of migrated and invaded cells in the oe-AKT1-JSD group was significantly less than that in cells of the oe-AKT1-control group. Moreover, the difference between the oe-AKT1-control and oe-AKT1-JSD groups was larger than that between the NC-control and NC-JSD groups. Compared with NC-control group, ***P<0.001; compared with oe-AKT1-control group, ●●●*P*<0.01; compared between NC-groups and oe-AKT1-groups, ▼▼▼*P*<0.001; n=9.

Wound healing assays were performed with SW480 and SW620 cells in four cell groups (NC-control, NC-JSD, oe-AKT1-control and oe-AKT1-JSD). All groups received EGF at a final concentration of 50 ng/ml for 48 h to induce EMT. NC-JSD and oe-AKT1-JSD groups received 6 mg/ml JSD treatment for an additional 48 h. As shown in Figure 8A-8B, the 24 h migration distance of both NC-JSD cell lines was significantly shorter than that of NC-control cells (both, P < 0.001), indicating that JSD treatment inhibited the healing ability of colon cancer cells. The 24 h migration distance of oe-AKT1-control cells was significantly longer than that of NC-control cells (both, P < 0.001), indicating that overexpression of AKT1 could enhance the healing ability of colon cancer cells. Additionally, the 24 h migration distance of cells in the oe-AKT1-JSD group was significantly shorter than that of oe-AKT1-control cells (both, P < 0.001). The difference between the oe-AKT1-control and oe-AKT1-JSD groups was larger than that between the NC-control and NC-JSD groups (both, P < 0.001), suggesting that the ability of JSD to inhibit the healing ability of colon cancer cells was enhanced after AKT1 overexpression.

Transwell assays were performed to assess the migration and invasion potential of both cell lines, with drug treatment the same as that done in wound healing assays (Figure 8C-8F). The number of migrated and invaded cells in the NC-JSD group was significantly lower than that in the NC-control group (all, P < 0.001), indicating that JSD dramatically inhibited the migration and invasion potential of colon cancer cells. In addition, the number of migrated and invaded cells in the oe-AKT1-control group was significantly higher than that in the NC-control group (all, *P* < 0.001), indicating that AKT1 overexpression could enhance the migration and invasion potential of colon cancer cells. Additionally, the number of migrated and invaded cells in the oe-AKT1-JSD group was significantly lower than that in cells of the oe-AKT1-control group (all, *P* < 0.001). Moreover, the difference between in the oe-AKT1-control and oe-AKT1-JSD groups was more than that between the NC-control and NC-JSD groups (both, *P* < 0.001), suggesting that the ability of JSD to inhibit the migration and invasion of colon cancer cells was enhanced after AKT1 overexpression.

### 3.7 Overexpression of AKT1 enhanced JSD-induced reversion of EMT *in vitro*

**Figure 9:**
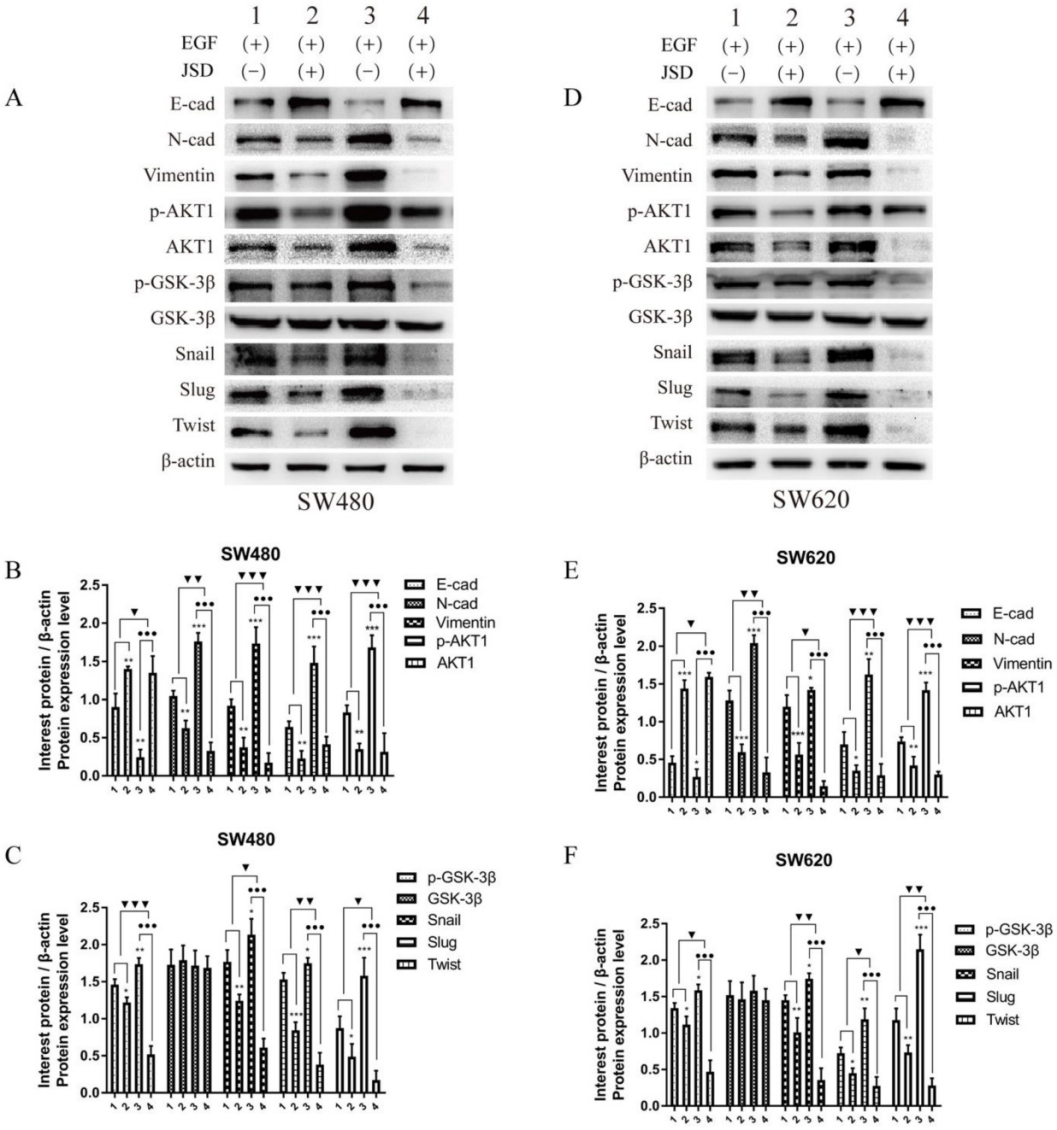
Overexpression of AKT1 enhanced JSD-induced reversion of EMT *in vitro.* JSD drug treatment for Western blot assays was similar to that done in wound healing assays (1=NC-control, 2= NC-JSD, 3=oe-AKT1-control, 4=oe-AKT1-JSD group). (A-C) The expression of indicated proteins in SW480 cells. (D-F) The expression of indicated proteins in SW620 cells. The expression of E-cadherin in both cell lines was markedly upregulated in the NC-JSD groups compared to that in the NC-control groups, whereas N-cadherin, vimentin, p-AKT1, AKT1, p-GSK-3β, GSK-3β, Snail, Slug and Twist expression was markedly downregulated. In addition, the expression of E-cadherin in both cell lines was markedly downregulated in the oe-AKT1-control groups compared to that in the NC-control groups, whereas N-cadherin, vimentin, p-AKT1, AKT1, p-GSK-3β, GSK-3β, Snail, Slug and Twist expression was markedly upregulated. The expression of E-cadherin in both cell lines was markedly upregulated in the oe-AKT1-JSD groups compared to that in the oe-AKT1-control groups, whereas N-cadherin, vimentin, p-AKT1, AKT1, p-GSK-3β, GSK-3β, Snail, Slug and Twist expression was markedly downregulated. Moreover, the difference between the oe-AKT1-control and oe-AKT1-JSD groups was larger than that between the NC-control and NC-JSD groups. Compared with NC-control group, *P<0.05, **P<0.01, ***P<0.001; compared with oe-AKT1-control group, ●●●*P*<0.001; compared between NC-groups and si-AKT1-groups, ▼*P*<0.05, ▼▼*P*<0.01, ▼▼▼*P*<0.001; n=3.

JSD drug treatment for Western blot assays was similar to that done in wound healing assays. As shown in Figure 9, the expression of E-cadherin in both cell lines was markedly upregulated in the NC-JSD groups compared to that in the NC-control groups, whereas N-cadherin, vimentin, p-AKT1, AKT1, p-GSK-3β, GSK-3β, Snail, Slug and Twist expression was markedly downregulated (all, P < 0.05), indicating that EMT status could be reversed after JSD treatment. In addition, the expression of E-cadherin in both cell lines was markedly downregulated in the oe-AKT1-control groups compared to the NC-control groups, whereas N-cadherin, vimentin, p-AKT1, AKT1, p-GSK-3β, GSK-3β, Snail, Slug and Twist expression was markedly upregulated (all, P < 0.05), indicating that overexpression of AKT1 can promote EMT. Furthermore, the expression of E-cadherin in both cell lines was markedly upregulated in the oe-AKT1-JSD groups compared to the oe-AKT1-control groups, whereas N-cadherin, vimentin, p-AKT1, AKT1, p-GSK-3β, GSK-3β, Snail, Slug and Twist expression was markedly downregulated (all, P < 0.001). Moreover, the difference between in the oe-AKT1-control and oe-AKT1-JSD groups was significantly larger than that between the NC-control and NC-JSD groups (both, *P* < 0.05), suggesting that the ability of JSD to reverse EMT status in colon cancer cells was enhanced by AKT1 overexpression.

### 3.8 JSD-induced inhibition on colon cancer liver metastasis *in vivo*

**Figure 10:**
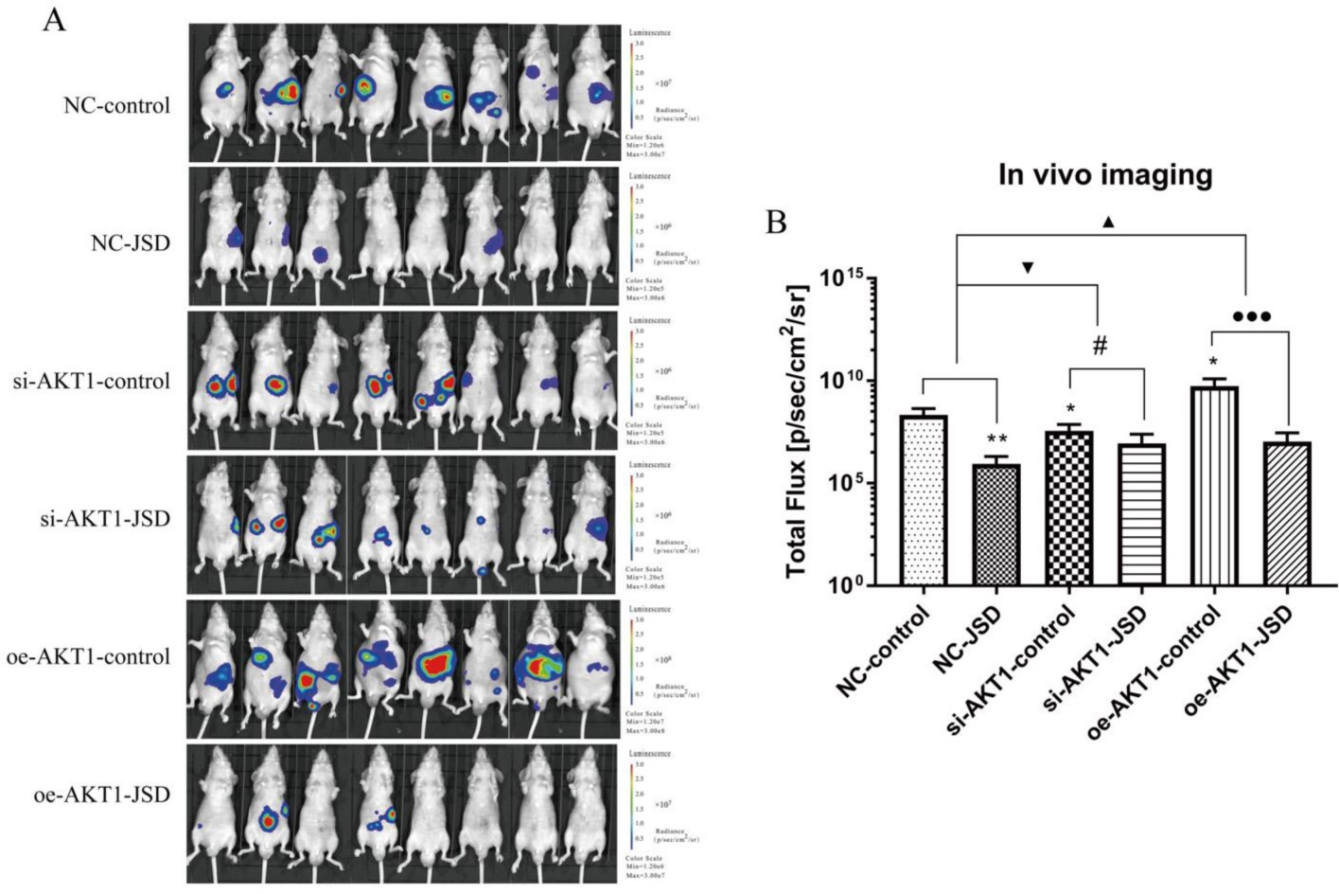
JSD-induced inhibition on colon cancer liver metastasis *in vivo.* Colon cancer liver metastasis of mice (NC-control, NC-JSD, si-AKT1-control, si-AKT1-JSD, oe-AKT1-control and oe-AKT1-JSD group) was observed by living fluorescence imaging technique. (A) Images with fluorescence signal in living mice. (B) The total flux of fluorescence signal in mice. Compared with NC-control group, *P<0.05, **P<0.01; compared with si-AKT1-control group, #*P*<0.05; compared with oe-AKT1-control group, ●●●*P*<0.001; compared between NC-groups and si-AKT1-groups, ▼*P*<0.05; compared between NC-groups and oe-AKT1-groups, ▲*P*<0.05; n=8.

We used living fluorescence imaging to evaluate liver metastasis in mice. As shown in Figure 10A and 10B, the total flux of mice was significantly decreased after JSD intervention or AKT1 silencing (NC-JSD, *P*<0.01; si-AKT1-control, *P*<0.05), while markedly increased after AKT1 overexpression (*P*<0.05). The difference in flux between the NC-JSD and NC-control mice was reduced when AKT1 was silenced. Conversely, when AKT1 was overexpressed, the difference between the control and JSD mice was enhanced (*P*<0.05). The above results suggest that liver metastasis of colon cancer cells can be significantly inhibited by JSD intervention or AKT1 knockdown, while significantly enhanced by AKT1 overexpression. The inhibitory effects of JSD on liver metastasis of colon cancer cells was significantly weakened when AKT1 was knocked down, while enhanced with overexpression of AKT1 (P<0.001). These results indicate that JSD-induced inhibition effects on liver metastasis of colon cancer cells is dependent upon levels of AKT1 expression.

**Figure 11:**
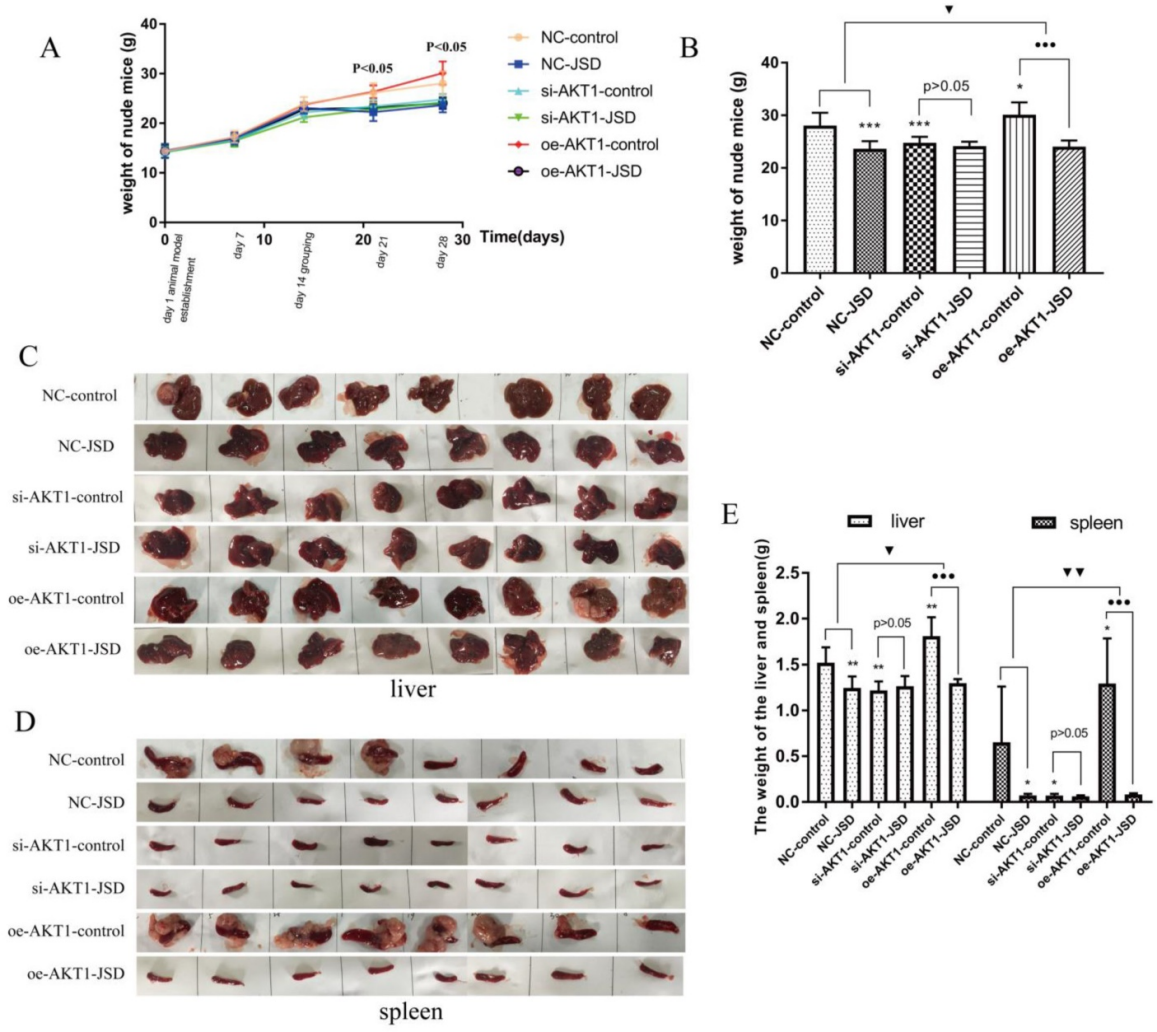
JSD-induced changes of mice body, liver and spleen weight *in vivo.* Mice (NC-control, NC-JSD, si-AKT1-control, si-AKT1-JSD, oe-AKT1-control and oe-AKT1-JSD) were prepared as described in the methods. (A) The body weights of mice after JSD-intervention on day 1, 7, 14, 21 and 28. (B) The body weights of mice on day 28. (C) Pictures of livers from mice. (D) Pictures of spleens from mice. (E) Liver and spleen weights. Compared with NC-control group, *P<0.05, **P<0.01, ***P<0.001; compared with oe-AKT1-control group, ●●●*P*<0.001; compared between NC-groups and oe-AKT1-groups, ▼*P*<0.05, ▼▼*P*<0.01; n=8.

Following JSD intervention, the weights of all mice were recorded until day 28. As shown in Figure 11A, by day 21 there was a difference in mouse weight between the six groups (NC-control, NC-JSD, si-AKT1-control, si-AKT1-JSD, oe-AKT1-control, oe-AKT1-JSD) and by day 28, oe-AKT1-control mice had the heaviest weight due to ascites, large tumor load, and metastatic weight. The weight of NC-control mice was also increased due to ascites in some of the mice in this group (Figure 11A and 11B). We observed that the weight of mice was significantly decreased after JSD intervention or AKT1 silencing (NC-JSD, P<0.001; si-AKT1-control, P<0.001), while markedly increased after AKT1 overexpression (P<0.05). There was no significant difference in weight between si-AKT1-JSD and si-AKT1-control groups, while the difference between NC-JSD and NC-control groups was enlarged with overexpression of AKT1 (P<0.05).

Liver metastases classified as giant block, small block and diffuse metastasis. To examine the degree of liver metastases, liver and spleen weight was continuously analyzed among the above six groups (Figure 11C-11E). Mice in oe-AKT1-control group had the heaviest liver or spleen weight due to massive metastatic tumors. NC-control mice also had an increase in liver or spleen weight due to small block and diffuse metastatic tumors. There was no significant difference in the liver or spleen weight of the remaining 4 groups. These results indicated that the liver metastasis of colon cancer can be markedly inhibited by JSD intervention or AKT1 knockdown, while significantly enhanced by AKT1 overexpression. The inhibitory effects of JSD on colon cancer liver metastasis can be significantly weakened or even completely abrogated with AKT1 knockdown, while obviously enhanced with AKT1 overexpression (P<0.05). Therefore, we concluded that the JSD-induced inhibition effects on colon cancer liver metastasis were realized by AKT1/GSK-3β signaling activation.

### 3.9 JSD-induced reversion of EMT depends on activation of AKT/GSK-3β signaling *in vivo*

**Figure 12:**
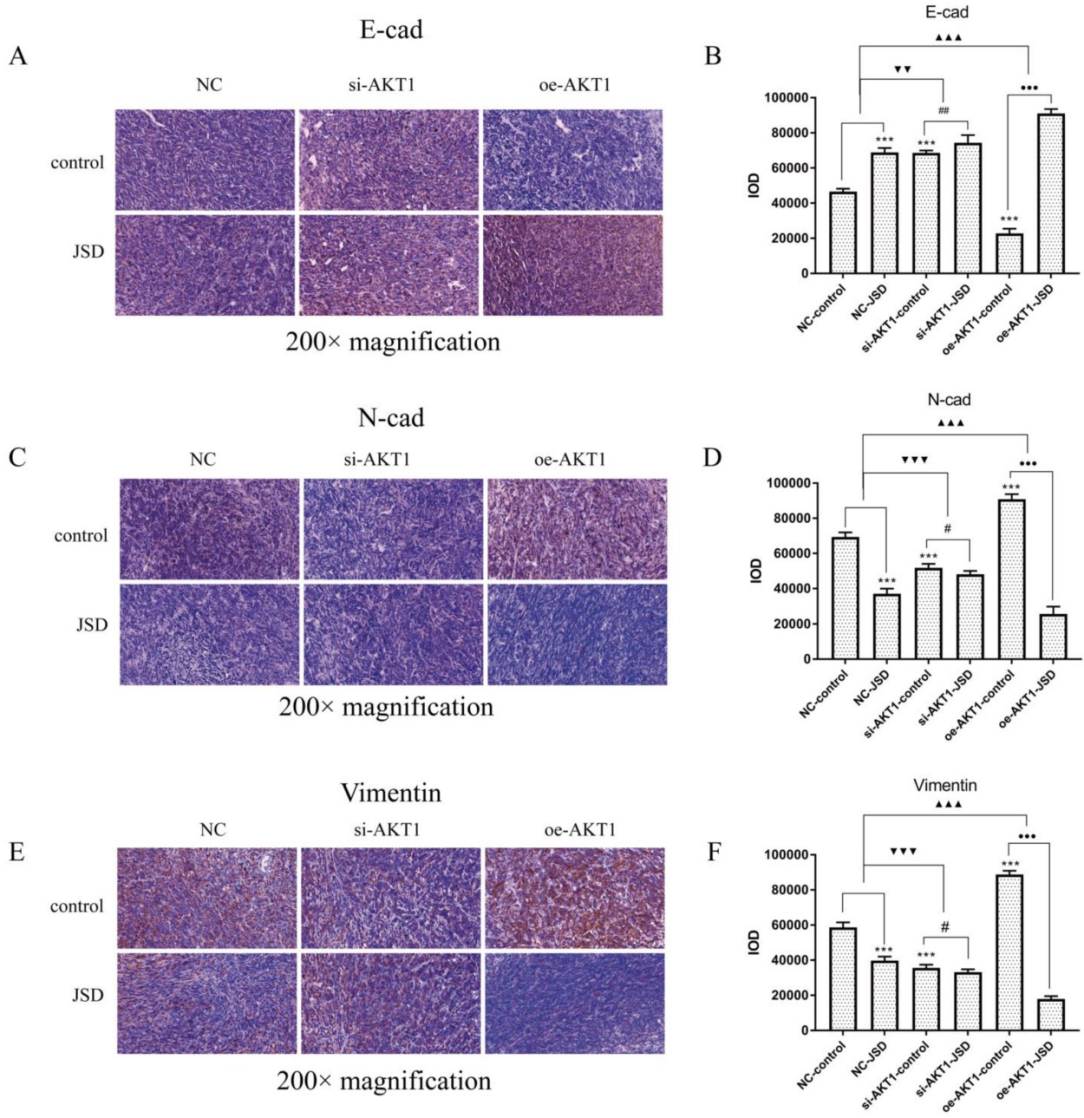
JSD-induced reversion of EMT depends on activation of AKT/GSK-3β signaling *in vivo.* Immunohistochemical staining was performed in tumor foci of mice to detect the expressions of E-cad, N-cad, and Vimentin. Pictures of immunohistochemical staining of E-cad (A), N-cad (C) and Vimentin (E) are shown. (B) The IOD of E-cad stain. (D) The IOD of N-cad stain. (F) The IOD of Vimentin stain. Compared with NC-control group, ***P<0.001; compared with si-AKT1-control group, #*P*<0.05, ##*P*<0.01; compared with oe-AKT1-control group, ●●●*P*<0.001; compared between NC-groups and si-AKT1-groups, ▼▼*P*<0.01, ▼▼▼*P*<0.001; compared between NC-groups and oe-AKT1-groups, ▲▲▲*P*<0.001; n=6.

Immunohistochemical staining was performed to observe the expression of E-cad, N-cad and Vimentin in tumor foci (Figure 12). Increased E-cad expression, as well as decreased N-cad and Vimentin expression, can be detected after JSD intervention or AKT1 silencing (P<0.001), which was opposite to that seen after overexpression of AKT1. The difference between the expression of E-cad, N-cad, and Vimentin between the NC-JSD and NC-control groups is weakened with knockdown of AKT1 and enhanced with overexpression of AKT1 (*P*<0.01).

**Figure 13:**
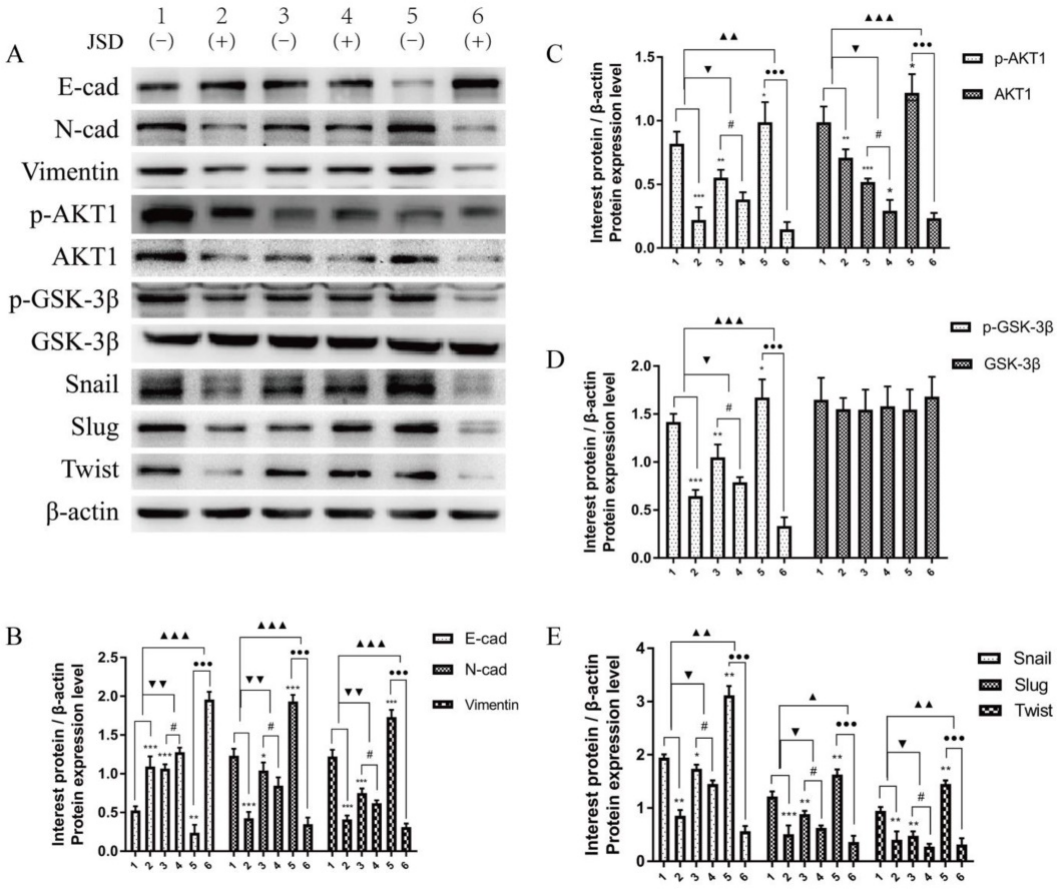
JSD-induced reversion of EMT depends on activation of AKT/GSK-3β signaling *in vivo.* WB assays were performed in tumor foci of mice to detect the expressions of E-cad, N-cad, Vimentin, p-AKT1, AKT1, p-GSK-3β, GSK-3β, Snail, Slug and Twist. (A) Expressions of indicated proteins in tumor foci of mice (1=NC-control, 2=NC-JSD, 3=si-AKT1-control, 4=si-AKT1-JSD, 5=oe-AKT1-control, 6=oe-AKT1-JSD). (B) Relative expression of E-cad, N-cad and Vimentin. (C) Relative expression of p-AKT1 and AKT1. (D) Relative expression of p-GSK-3β and GSK-3β. (E) Relative expression of Snail, Slug and Twist. Compared with NC-control group, *P<0.05, **P<0.01, ***P<0.001; compared with si-AKT1-control group, #*P*<0.05; compared with oe-AKT1-control group, ●●●*P*<0.001; compared between NC-groups and si-AKT1-groups, ▼*P*<0.05, ▼▼*P*<0.01; compared between NC-groups and oe-AKT1-groups, ▲*P*<0.05, ▲▲*P*<0.01, ▲▲▲*P*<0.001; n=3.

To verify the EMT-related protein (E-cad, N-cad, Vimentin, Snail, Slug and Twist) and AKT1/GSK-3β signaling pathway associated protein expressions *in vivo*, Western Blot assays were performed in tumor tissues of mice. As shown in Figure 13, the expression changes of these proteins in colon tumor tissues were in accordance with that in colon cancer cells. These results indicate that EMT of colon cancer can be significantly reversed by JSD intervention or AKT1 knockdown, while significantly enhanced by AKT1 overexpression. Additionally, JSD-induced reversion of colon cancer EMT can be significantly weakened with knockdown of AKT1 and enhanced with overexpression of AKT1. Therefore, it can be concluded that JSD-induced reversion of EMT in colon cancer is realized by the activation of AKT1/GSK-3β signaling.

## 4. Discussion

Metastatic CRC is a heterogeneous disease with a poor prognosis in advanced stages. The global burden of CRC is expected to increase by 60% by 2030^20^. Besides common therapies such as surgery, chemotherapy and radiotherapy, TCM has been a novel additional method for CRC treatment^21^. According to TCM, the main pathogenesis of CRC is intestines embedded with heat and toxin^21^. Therefore, clearing away heat and toxic materials is the main approach to curing CRC. JSD is an empirical Chinese medicine used for removing heat and toxic materials and has been used by Traditional Chinese Medical Hospital of Zhejiang Province for colon cancer treatment. In our prospective study we have reported that JSD can increase the 1 year survival rate of CRC patients by 8.47% and the 2 year survival rate by 19.38% and increase the curative effect of CRC chemotherapy and improve the life quality of CRC patients (data to be published). Therefore, it is important to study the mechanism of JSD treatment in depth.

In this study, we found that JSD could inhibit the migration and invasion of colon cancer cells, and could reverse the EMT status of colon cancer cells through the AKT/GSK-3β signaling pathway. We successfully constructed AKT1 knockdown (si-AKT1) and overexpression (oe-AKT1) cells in both SW480 and SW620 cell lines. We then found that AKT/GSK-3β signaling is necessary for JSD to exhibit its anti-cancers effects. For EMT reversion and metastasis inhibition in colon cancer cells, JSD has weaker effects in AKT1-silenced cells and more powerful effects in AKT1-overexpressed cells, which can also be observed in corresponding *in vivo* studies. Taken together, these data suggest that JSD can reverse EMT and inhibit colon cancer metastasis through the AKT/GSK-3β signaling pathway *in vitro* and *in vivo*.

EMT is a complex process of phenotypic transformation between epithelial cells and interstitial cells^22^, which is closely related to invasion and metastasis of tumor cells^23^ Downregulation of E-cad and upregulation of N-cad and Vimentin are important characteristics of EMT^24^. EMT is regulated by multiple transcription factors, including zinc finger protein family (Snail, Slug), bHLH transcription family (Twist, E47), ZEB family (ZEB1, ZEB2), etc^25^. Snail/Slug can compete with Smad interacting protein (Sip1) to bind the E-box sequence of the E-cad promoter region, then inhibit E-cad expression and promote Vimentin expression to induce EMT^26^. Snail can also mediate E-cad repression to induce EMT through recruiting the Sin3A/histone deacetylase 1 (HDAC1)/HDAC2 complex^27^. In addition, abnormal Twist1 expression can lead to E-cad downregulation or deletion, resulting in cell adhesion loss and promoting EMT^28^. Other studies have revealed that Twist1 can down-regulate E-cad and up-regulate N-cad to trigger EMT by increasing methyltransferase SET8 level and regulating H4K20 monomethylation activity^29^. Our study has revealed that JSD can significantly upregulate E-cad and downregulate N-cad, Vimentin, Snail, Slug and Twist1 in colon cancer cells, leading to a reversion of EMT.

Accumulating evidence has uncovered that activation of the PI3K/AKT axis is an important feature of EMT^30^, ^31^. AKT has been reported to suppress the transcription of E-cad, which induces cellular responses leading to the conversion of epithelial cells into invasive mesenchymal cells and tumor metastasis^32^. Therefore, it is relevant to investigate AKT's involvement in JSD-induced reversion of EMT and inhibition of metastasis in colon cancer. Our study found that the inhibition effects of JSD on colon cancer EMT and metastasis were weakened with knockdown of AKT1 and inactivation of AKT/GSK-3β signaling. Conversely, overexpression of AKT1 and activation of the AKT/GSK-3β signaling pathway enhanced JSD's ability to inhibit EMT and colon cancer metastasis. These data suggest that AKT/GSK-3β signaling is crucial for JSD to exert its effects in the context of colon cancer. However, although the effects of JSD were weakened with AKT1 knockdown, inhibition of EMT and metastasis was not completely abrogated, suggesting that JSD may also work through other signaling pathways. This finding provides more evidence that TCM works through complex mechanisms involving multiple pathways, targets and links.

Multiple evidence has shown that TCM can regulate EMT through the PI3K/AKT signaling pathway in human tumors. For example, berberine can suppress EMT in melanoma cells by regulating cross-talk between PI3K/AKT and RARα/RARβ signaling^33^. Additionally, litchi seed extracts can halt prostate cancer progression via induction of apoptosis and attenuation of EMT through AKT/GSK-3β signaling^34^. Furthermore, oridonin can prevent migration, invasion, cell adhesion, and TGF-β1-induced EMT in melanoma cells by inhibiting the activity of the PI3K/AKT/GSK‑3β signaling pathway^35^. However, the discovery that JSD can reverse EMT and inhibit colon cancer invasion and metastasis through AKT/GSK-3B signaling is a novel finding. JSD is a mixed extractive and to further elucidate its mechanism of action future studies need to be done to determine which component is responsible for its antitumor effects. This information will provide a better theoretical foundation for the development and clinical application of TCM.

## 5. Conclusions

In summary, JSD may downregulate the EMT transcription factors Snail, Slug and Twist through the AKT/GSK-3β signaling pathway, leading to an upregulation of E-cad, and downregulation of N-cad and Vimentin, reversing EMT and inhibiting invasion and metastasis of colon cancer cells. The data also suggests that the AKT/GSK-3β signaling pathway is crucial for JSD to exert its effects on EMT.

## Supplementary Material

Supplementary figures and tables.

## Figures and Tables

**Table 1:** Proliferation inhibition rates (IR) of SW480, SW620 and HCT-8 cell lines treated with various concentrations of JSD for 48 h (X±S, n=9).

Concentration (mg/ml)	0	1	2	4	8	16	32	64
IR(% ) in SW480	1.31±4.17	1.83±4.06	13.46±2.69	32.91±5.38	59.12±6.46	79.58±4.07	88.58±5.87	95.35±2.84
IR(% ) in SW620	0.06±6.48	1.45±4.57	10.85±2.83	28.36±7.12	49.64±3.59	78.15±3.87	90.73±3.09	95.88±2.61
IR(% ) in HCT-8	-0.22±4.42	3.25±4.41	15.05±3.78	33.87±3.04	57.56±4.27	85.69±3.42	91.03±2.60	97.13±2.65

## Abbreviations

JSD: Jiedu Sangen Decoction
EMT: Epithelial-mesenchymal transformation
CRC: Colorectal cancer
TCM: Traditional Chinese Medicine
E-cad: E-cadherin
N-cad: N-cadherin
ZEB1: zinc-finger E-box binding homeobox 1
AKT: The serine/threonine kinase
EGF: epidermal growth factor
HCC: human colon cancer
FBS: fetal bovine serum
