# Jiedu Sangen Decoction Reverses Epithelial-to-mesenchymal Transition and Inhibits Invasion and Metastasis of Colon Cancer via AKT/GSK-3β Signaling Pathway: Erratum

**DOI:** 10.7150/jca.80633

**Published:** 2022-12-30

**Authors:** Li Yuan, Kai Zhang, Meng-Meng Zhou, Harpreet S. Wasan, Fang-Fang Tao, Qing-Ying Yan, Guan Feng, Yin-Shan Tang, Min-He Shen, Sheng-Lin Ma, Shan-Ming Ruan

**Affiliations:** 1 The First Clinical Medical College of Zhejiang Chinese Medical University, Hangzhou, 310053, Zhejiang, china; 2 Department of Medical Oncology, The First Affiliated Hospital of Zhejiang Chinese Medical University, Hangzhou, 310006, Zhejiang, China; 3 Department of traditional Chinese medicine, The First people's Hospital of Quzhou, 324000, Zhejiang, China; 4 Department of Cancer Medicine, Hammersmith Hospital, Imperial College Healthcare NHS Trust, London, W12 0HS, UK; 5 Department of Immunology and Microbiology, Basic Medical College, Zhejiang Chinese Medical University, Hangzhou, 310053, Zhejiang, China; 6 Department of Rehabilitation in Traditional Chinese Medicine, The Second Affiliated Hospital of Zhejiang University School of Medicine, Hangzhou, 310009, Zhejiang, China; 7 Department of Oncology, The Forth Affiliated Hospital of Zhejiang Chinese Medical University, Hangzhou, 310006, Zhejiang, China

Recently, we sorted out all the original data and found that several figures in our article have errors during image assembly. The corrected figures are provided below. We are very sorry for the errors caused by our negligence.

## Figures and Tables

**Figure 3D:**
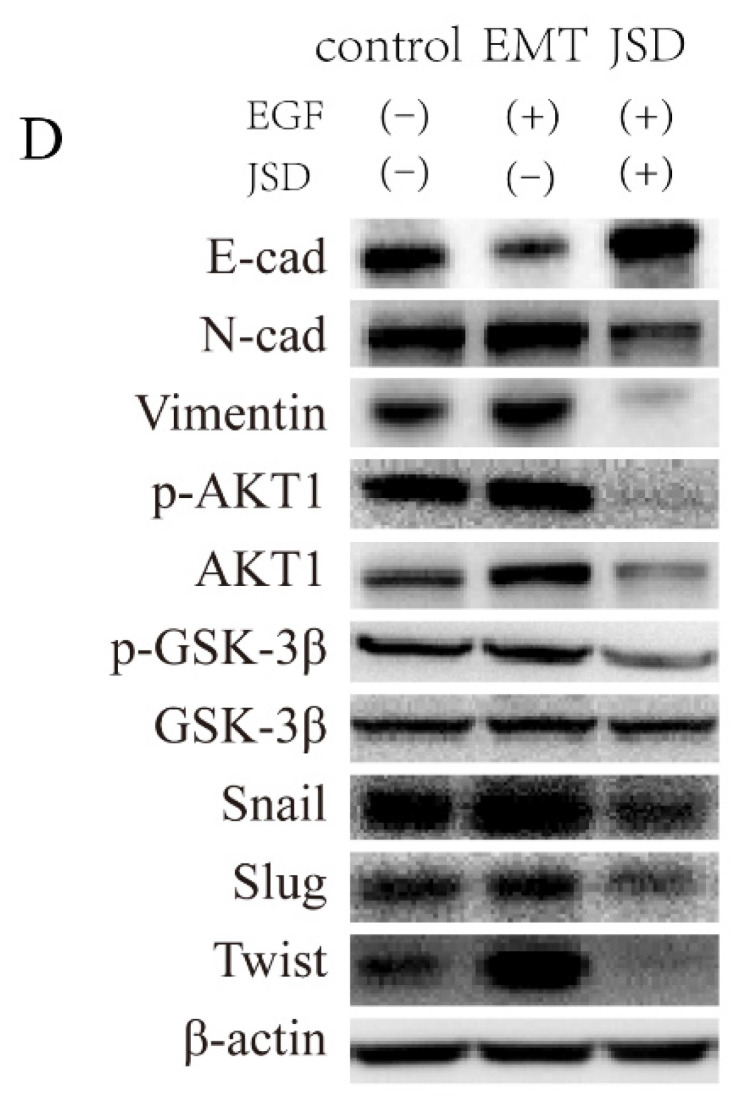
Correct image.

**Figure 5:**
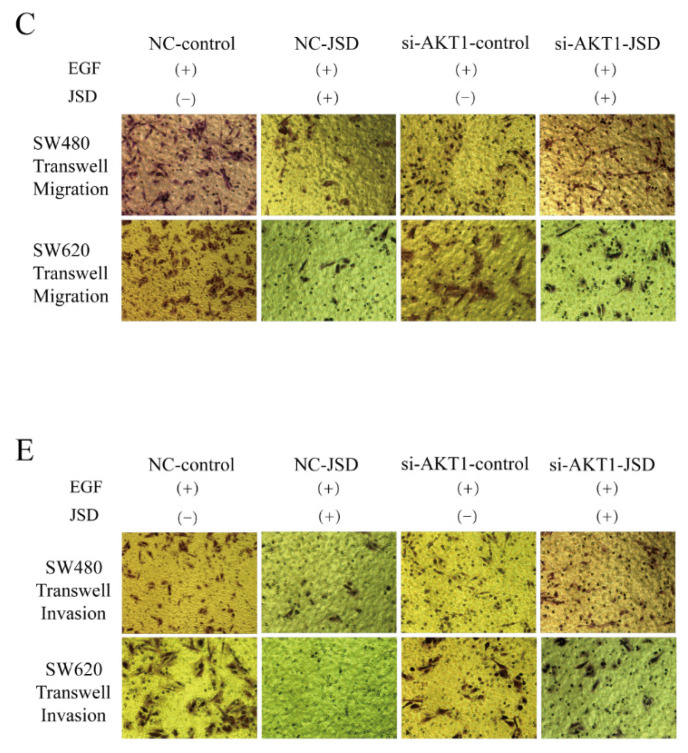
Correct image.

**Figure 6:**
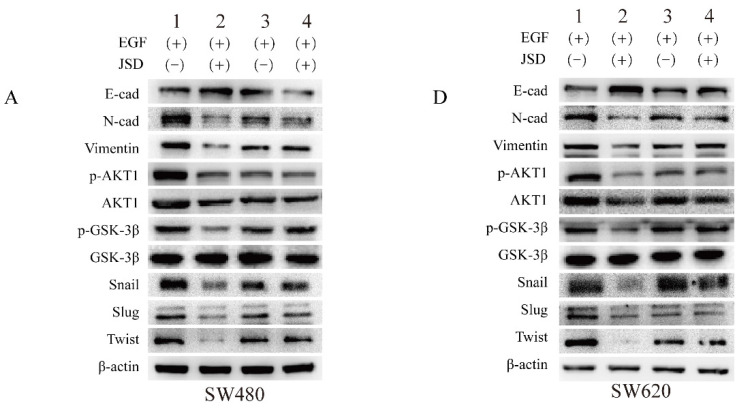
Correct image.

**Figure 9:**
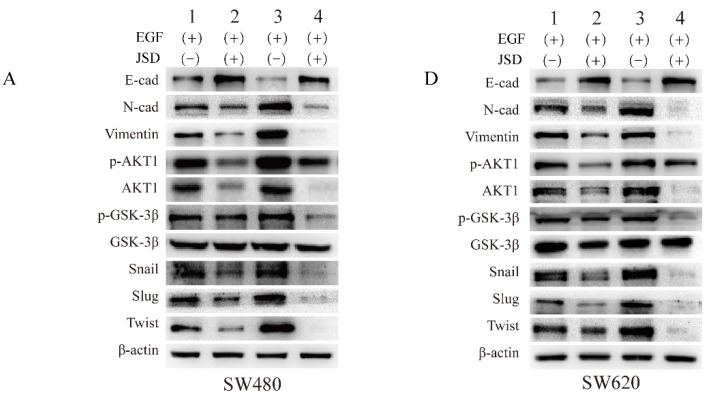
Correct image.

**Figure 12:**
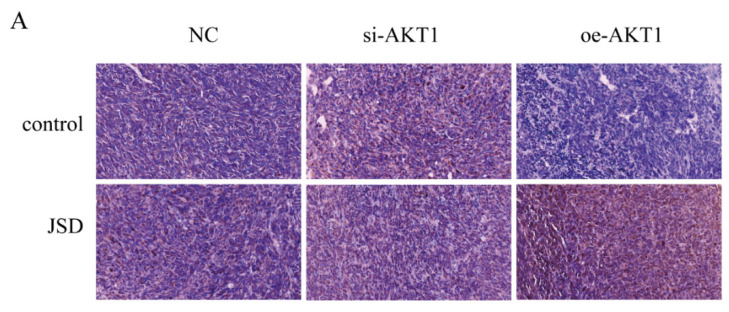
Correct image.

