# Supplementary material for: Jiedu Sangen Decoction Reverses Epithelial-to-mesenchymal Transition and Inhibits Invasion and Metastasis of Colon Cancer via AKT/GSK-3β Signaling Pathway
Source: J Cancer. 2019 Oct 20;10(25):6439–56. doi: 10.7150/jca.32873 (PMC6856737; doi:10.7150/jca.32873)
Supplement: Supplementary file 1 — Supplementary figures and tables. [file jcav10p6439s1.pdf]

Supplementary Material

Jiedu Sangen Decoction Reverses Epithelial-to-mesenchymal Transition and Inhibits Invasion and Metastasis of Colon Cancer via AKT/GSK-3β Signaling Pathway

Li Yuan[1], Kai Zhang[2], Meng-Meng Zhou[3], Harpreet S. Wasan[4], Fang-Fang Tao[5], Qing-Ying Yan[1], Guan Feng[1], Yin-Shan Tang[6], Min-He Shen[2], Sheng-Lin Ma[7], Shan-Ming Ruan[2,7]*

1 Supplementary Figures and Tables

1.1 Supplementary Figures

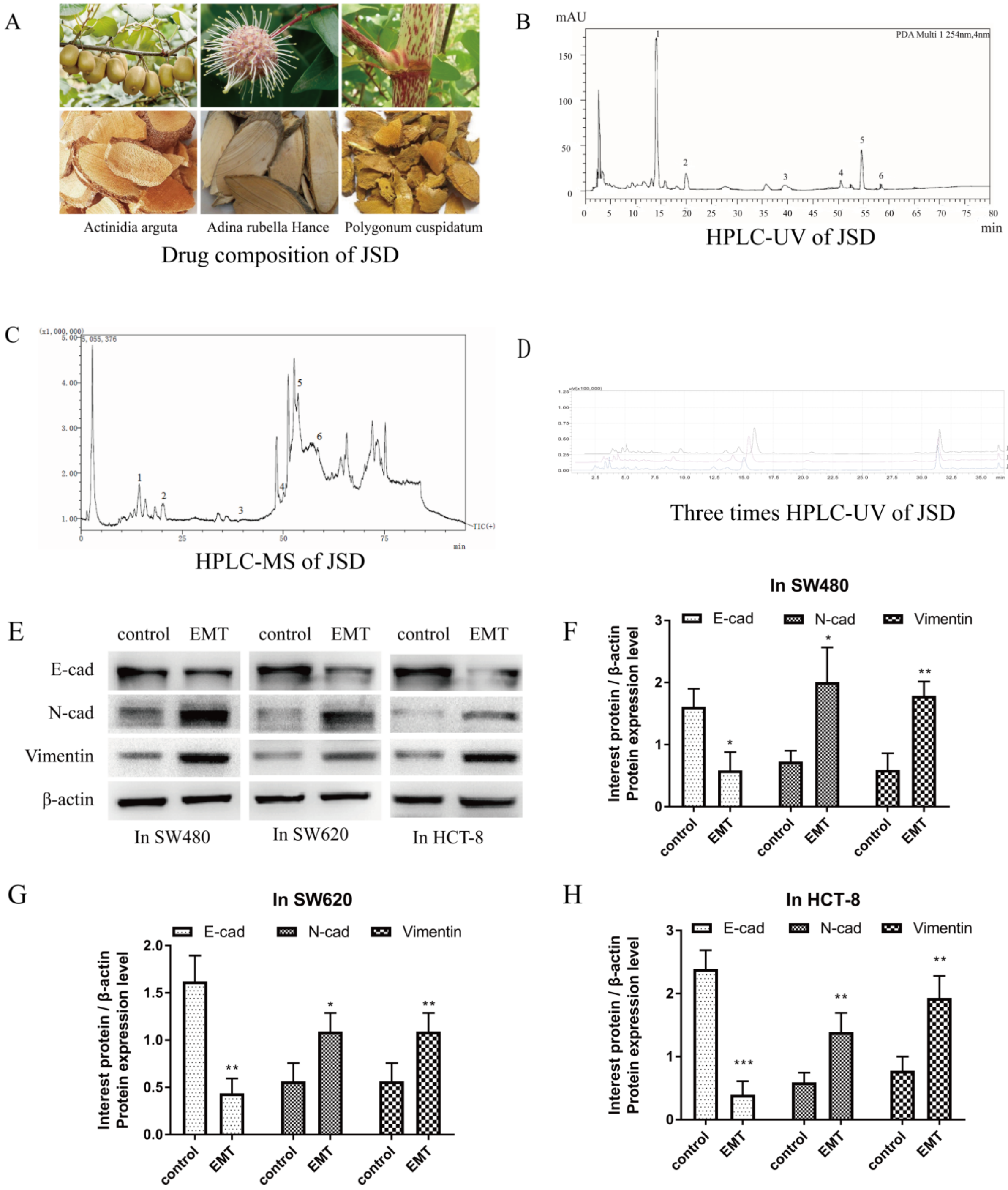

Fig.1S. The composition and preparation of JSD and the success of EGF-induced EMT model in colon cancer cells. (A) Drug composition of JSD. (B) HPLC-UV of JSD. (C) HPLC-MS of JSD. (D) Three time HPLC-UV of JSD. Cells were treated with or without EGF, and then divided into EMT and control group, respectively. (E) Expressions of E-cad, N-cad and Vimentin in SW480, SW620 and HCT-8 cells. (F) Relative expression of E-cad, N-cad and Vimentin in SW480 cells. (G) Relative

expression of E-cad, N-cad and Vimentin in SW620 cells. (H) Relative expression of E-cad, N-cad and Vimentin in HCT-8 cells. Compared with the control group, **P*<0.05, ***P*<0.01, ****P*<0.001; n=3.

1.2 Supplementary Tables

Table1 The LC-MS Component Analysis of JSD

No.	Retention time	Molecular weight	Molecular formula	Potential substance	Corresponding plants
1	14.050	486	$C_{21}H_{26}O_{13}$	5-hydroxy-2-methylchromone-7-O-β-D-xylopyranosyl(1→6)-β-D-glucopyranoside	Adina fauriei H.Lév
2	19.883	390	$C_{20}H_{22}O_{8}$	Resveratrol-3-O-glucoside	Polygonum cuspidatum Siebold & Zucc.
3	39.367	390	$C_{24}H_{38}O_{4}$	Di-(2-ethylthexyl) phthalate	Actinidia argute Siebold & Zucc
4	50.433	390	$C_{20}H_{22}O_{8}$	Resveratrol-4'-O-glucoside	Polygonum cuspidatum Siebold & Zucc.
5	54.600	432	$C_{21}H_{20}O_{10}$	Emodin-1-O-beta-D-glucopyranoside	Polygonum cuspidatum Siebold & Zucc.
6	58.483	446	$C_{22}H_{22}O_{10}$	Emodin methyl ether-8-O-beta-D-glucoside	Polygonum cuspidatum Siebold & Zucc.

Table1. The place of origin of all three materials is Zhejiang. Actinidia argute Siebold & Zucc. (100 g), Adina fauriei H.Lév. (100 g), and Polygonum cuspidatum Siebold & Zucc. (100 g) were mixed and immersed in 1000 mL of distilled water for 30 min. The filtrates were concentrated to 150 mL to obtain JSD such that its crude drug content was 2 g/mL of mother liquor. The JSD was prepared at the China Pharmaceutical University (Jiangsu, China). Through the information analysis of HPLC-UV and HPLC-MS for JSD, the quality control indicator is confirmed as di-(2-ethylthexyl) phthalate

for Actinidia argute Siebold & Zucc., 5-hydroxyl-2-methyl chromocone-7-O-beta-D-celery sugar (1 to 6)-beta-D-glucoside for Adina fauriei H.Lév., and Resveratrol-3-O-glucoside for Polygonum cuspidatum Siebold & Zucc.
